# Inflammasome activity regulation by PUFA metabolites

**DOI:** 10.3389/fimmu.2024.1452749

**Published:** 2024-09-03

**Authors:** Sinemyiz Atalay Ekiner, Agnieszka Gęgotek, Elżbieta Skrzydlewska

**Affiliations:** Department of Analytical Chemistry, Medical University of Bialystok, Bialystok, Poland

**Keywords:** oxidative stress, inflammation, lipid mediators, PUFA metabolites, eicosanoids, inflammasome complexes

## Abstract

Oxidative stress and the accompanying chronic inflammation constitute an important metabolic problem that may lead to pathology, especially when the body is exposed to physicochemical and biological factors, including UV radiation, pathogens, drugs, as well as endogenous metabolic disorders. The cellular response is associated, among others, with changes in lipid metabolism, mainly due to the oxidation and the action of lipolytic enzymes. Products of oxidative fragmentation/cyclization of polyunsaturated fatty acids (PUFAs) [4-HNE, MDA, 8-isoprostanes, neuroprostanes] and eicosanoids generated as a result of the enzymatic metabolism of PUFAs significantly modify cellular metabolism, including inflammation and the functioning of the immune system by interfering with intracellular molecular signaling. The key regulators of inflammation, the effectiveness of which can be regulated by interacting with the products of lipid metabolism under oxidative stress, are inflammasome complexes. An example is both negative or positive regulation of NLRP3 inflammasome activity by 4-HNE depending on the severity of oxidative stress. 4-HNE modifies NLRP3 activity by both direct interaction with NLRP3 and alteration of NF-κB signaling. Furthermore, prostaglandin E2 is known to be positively correlated with both NLRP3 and NLRC4 activity, while its potential interference with AIM2 or NLRP1 activity is unproven. Therefore, the influence of PUFA metabolites on the activity of well-characterized inflammasome complexes is reviewed.

## 1 Introduction

The human organism is constantly exposed to various factors affecting systemic and cellular metabolism, including exogenous factors, such as UV radiation contained in sunlight, pathogens, and drugs, as well as endogenous factors, including those resulting from metabolic disorders at the level of the mitochondrial respiratory chain, endoplasmic reticulum, peroxisomes, and biological membranes, the components of which are metabolized into pro-oxidant and pro-inflammatory factors (1). Oxidative stress resulting from the above changes promotes the development of pathological states of the cellular microenvironment associated with chronic inflammation and further metabolic disorders, accompanied by changes in the dynamics of cell survival, such as those observed in the case of myocardial infarction, diabetes, and the development of cancer (2). Linking the causes and effects of oxidative stress and inflammation may contribute to the development of effective therapeutic strategies aimed at preventing and/or limiting emerging metabolic disorders. However, this requires a thorough analysis of the dynamic and complex interactions of factors involved in the development of oxidative stress and inflammation, as well as broadly understood metabolic consequences, both local and systemic.

**Figure 1:**
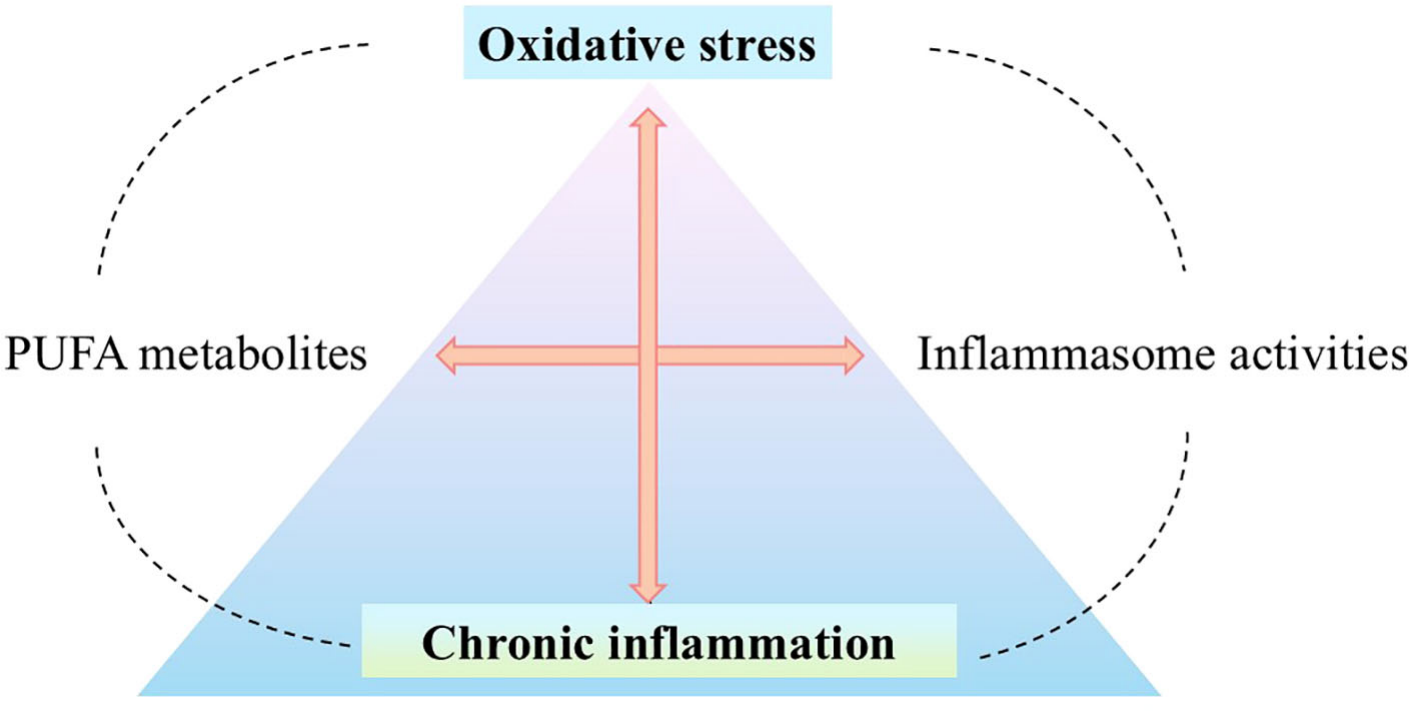
Oxidative stress and inflammation, dynamically interacting with each other (4, 5), may participate in the emergence and development of many diseases, such as cardiovascular diseases (6), neurodegenerative diseases (7), metabolic diseases (8), and cancer (9). Inflammatory cells - neutrophils and macrophages - release large amounts of ROS at the site of inflammation, such as O_2_^•−^, ^•^NO, ONOO-. They promote the generation of oxidative stress which is a critical regulator of the inflammatory response by inducing the production of inflammatory cytokines via expression of the pro-inflammatory genes (5, 10) as a result of modulation of signal transduction pathways such as NF-κB (7, 11). Therefore, in the place where oxidative stress and the associated chronic inflammation stand, the interference of PUFA metabolites with inflammasomes (directly/indirectly) is an important factor regulating the redox balance. (O_2_^•−^, superoxide anion; ^•^NO, nitric oxide; ONOO-, peroxynitrite; ROS, reactive oxygen species; RNS, reactive nitrogen species; NF-κB, nuclear factor - kappa B; PUFA, polyunsaturated fatty acid).

Considering the potential consequences of oxidative stress, multiprotein inflammasome complexes can be considered critical regulators of the inflammatory side of oxidative stress that drive the immune response and inflammation through proteolytic activation of pro-inflammatory caspases (3). Moreover, dynamic changes in PUFA metabolism resulting from oxidative stress appear to be a critical modulator of inflammation and immune function due to the immunomodulatory role of the mentioned lipid metabolites (**Figure 1**). PUFA metabolites can modulate the inflammatory response in many ways, including both anti-inflammatory and pro-inflammatory effects (12). The changes taking place are dynamic because they occur under the influence of numerous PUFA metabolites and their interactions with various intracellular receptors (both agonistic and antagonistic changes are observed in their action). So ultimately, they interfere with various metabolic pathways, concerning their balance and competitiveness on the cellular level, as well as the specificity of cells and their microenvironment (12, 13). Therefore, regulation of inflammasome activity, as part of the modulatory effects of PUFA metabolites, also appears to be crucial for understanding complex inflammatory signaling.

Therefore, understanding the effects of PUFA metabolites on the functions of inflammasome complexes is important to uncover the molecular dynamics behind oxidative stress and chronic inflammation. The work analyzes the influence of PUFA metabolites on the already well-characterized activity of inflammasomes. We hope that this may explain the complex molecular interactions underlying the strong communication between oxidative stress and associated chronic inflammation by PUFA metabolism. However, such an approach may contribute to the development of new therapeutic approaches aimed at pathologies associated with oxidative stress using antioxidant therapies, the limitations and failures of which have been indicated in various *in vivo* and clinical studies (14).

## 2 An emerging cellular response to oxidative stress: alteration of PUFA metabolism

Lipids, the main components of biological membranes (15), play an important structural (ensuring the polarity and permeability of biological membranes) and signaling roles in the molecular biology of the cell as well as constitute an energy reserve (16). These biologically important cellular components include phospholipids (such as phosphatidylglycerols and sphingomyelin), free fatty acids, and ceramides as well as mono-, di- and tri-acylglycerols, and sterols (17). Their chemical moieties are susceptible to oxidative modifications (17), and this situation makes them major targets of ROS and RNS in the case of redox biology. The structures of fatty acid acyl chains present the widest range of possible modifications and generated oxidation products (18).

This situation favors various lipid oxidation reactions accompanying oxidative stress, and more specifically, the formation of highly bioactive lipid peroxidation products, which are biomarkers of oxidative stress reflecting the severity of the intracellular oxidative state, as well as enzymatically generated metabolites of PUFAs which is intensified under the influence of oxidative stress. PUFAs are classified in omega-3 (ω-3) and omega-6 (ω-6) fatty acids such as arachidonic (AA), linoleic (LA), linolenic (ALA), eicosapentaenoic (EPA), and docosahexaenoic acids (DHA), containing more than one carbon-carbon double bonds, found in the structure of membrane phospholipids, glycolipids and cholesterol (16, 19, 20). They are very susceptible to oxidant attacks (16, 20). In lipid peroxidation, majorly PUFAs are oxidized (21). It is initiated by H_2_O_2_, metal transition ions Fe²^+^, Cu²^+^ (by H_2_O_2_ decomposition and homolysis of endogenous hydroperoxides) as well as peroxynitrite (22, 23) and a complex biochemical process takes place: the formation and propagation of lipid radicals, up taking oxygen, rearrangement of the double bonds in unsaturated lipids, and production of a variety of breakdown products, including alcohols, ketones, alkanes, aldehydes and ethers (24). Due to hydrogen abstraction from carbon, with oxygen insertion, lipid peroxyl radicals, and hydroperoxides are generated (16, 20, 25). Particularly, predominantly found ω-6 fatty acid (AA) can be reduced non-enzymatically to malondialdehyde (MDA), 4-hydroxy-2-nonenal (4-HNE), and the other lipid peroxidation end-products presenting stability and toxicity as well as high reactivity (16). Also, regarding to especially DHA, 8-isoprostanes and neuroprostanes are generated, through the propagation of oxidation chain reactions, following oxidative cyclization (26).

AA can be also enzymatically metabolized to several oxygenated derivatives (27). In general, it starts with the hydrolysis of membrane phospholipids by phospholipase A2 (PLA2s) into PUFAs, and next, their metabolism by cyclooxygenases (COXs), lipoxygenases (LOXs) and cytochromes p450 (CYP450s) follows (28). And, highly bioactive eicosanoids, including prostaglandins (PGs) and thromboxanes (TXs), leukotrienes (LTs), and lipoxins (LXs) and various epoxy, hydroxy and dihydroxy derivatives are generated (29). In particular, PGs and TXs are generated from the COX pathway; LTs, LXs and hydroxyeicosatetraenoic acids (HETEs), which are precursors for lipoxins, protectins, resolvins, and hydroxyoctadeca-dienoic acids (HODEs), are generated from the LOX pathways; and various epoxy, hydroxy and dihydroxy derivatives are produced via the CYP450s pathways (30, 31). Other than AA, various ω-3 and ω-6 PUFAs can be also eicosanoid precursors such as DHA, EPA, and LA (31).

Both of the above-mentioned PUFA metabolites play critical roles in cell functionality and survival by interfering with intracellular molecular signalization. These metabolites, being the results of lipid metabolism enhanced by oxidative stress, are known to cause cellular and tissue damage by damaging cell membranes and causing protein and nucleic acid covalent modifications (21), as seen also in the case of chronic inflammation (32–34). Redox-sensitive proteins (including ion transporters, receptors, signaling molecules, transcription factors, cytoskeletal structural proteins, and matrix metalloproteases) are reversibly oxidatively modified under normal physiological conditions (35). However, under pathological oxidative stress conditions, they are going to modify irreversibly and result in loss of protein function or protein aggregation, even disruption of intracellular redox signaling, and ultimately leading to cell and tissue damage. Thus, together with oxidative stress-mediated protein and DNA oxidative modifications (15, 36), PUFA metabolites induce several complex metabolic changes by altering cell biochemistry, intracellular signalization, and eventually cell functionality, and in this way, it can lead to pathophysiological consequences (36). There is a strong correlation between chronic deterioration in lipid metabolism, oxidative stress, and inflammation (37).

It is known that unsaturated aldehydes (such as 4-HNE, MDA), which are products of lipid peroxidation, can easily orchestrate DNA, and protein structural changes leading to alteration of membrane integrity and signal transduction, including gene expression of receptors, kinases, redox-sensitive transcription factors such as master cytoprotective Nrf2 (nuclear factor 2 associated with erythroid 2), responsible for proper cellular antioxidant action, and NF-κB, a key factor for inflammatory cellular response (16, 21, 38–40). DNA-protein crosslinks, formed by MDA interaction with proteins and nucleic acids, are involved in the regulation of the innate immune system through the expression of pro-inflammatory genes and the activation of several downstream inflammatory signaling pathways (41). However 4-HNE and 4-HNE-modified proteins are found to be involved in cell signaling by taking part in inflammatory reactions and participating in the progression of several chronic human diseases and systemic chronic inflammation (42, 43).

**Figure 2:**
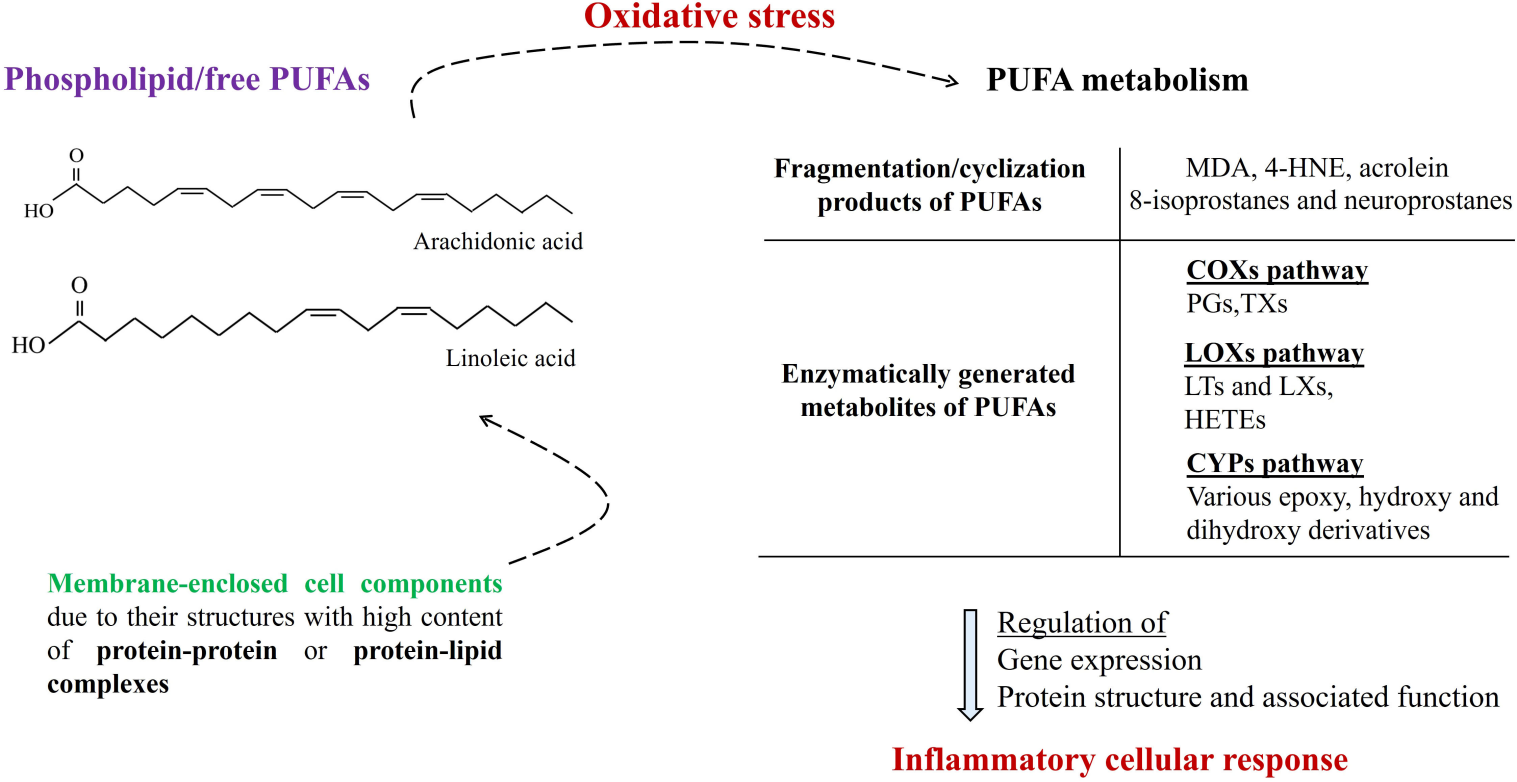
Membrane-enclosed cell components (such as nucleus, peroxisome, mitochondria, endoplasmic reticulum, and lysosome) due to their structures with high content of protein-protein or protein-lipid complexes dynamically participate in intracellular molecular signalization. Under conditions of oxidative stress, the generation of PUFA metabolites is increased, which significantly participates in the regulation of the inflammatory cellular response. (PUFAs, polyunsaturated fatty acids; MDA, malondialdehyde; 4-HNE, malondialdehyde; COXs, cyclooxygenases; LOXs, lipoxygenases; CYPs, cytochromes P-450s; PGs, prostaglandins: TXs, thromboxanes; LTs, leukotrienes; LXs, lipoxins; HETEs, hydroxyeicosatetraenoic acids).

Furthermore, enzymatically generated PUFA metabolites, such as eicosanoids and eicosanoid-related metabolites, including thromboxanes, prostaglandins, leukotrienes, and resolvins, critically participate in intracellular signaling, as the regulators of upstream activation of human systemic inflammation (44) which is synergistically contributing with oxidative stress (45). To give more precise examples: PGs can activate cell-surface G protein-coupled receptors (such as prostaglandin receptors (EPs) 1-4) and participate in the regulation of several biologically critical pathways such as NF-κB and MAP/ERK pathways; LXs (such as LXA4, LXB4) including down-regulation of acute inflammation enhance resolution via increasing monocyte chemotaxis; also some resolvins (such as RvE1) and epoxyeicosatrienoic acids take part of anti-inflammatory cellular actions (46). Moreover, due to its participation in the regulation of MAPK and PI3K/AKT signaling pathways, the therapeutic potential of LXA4 is underlined with a potential of fewer side effects comparing traditional anti-inflammatory approaches in cardiometabolic diseases (47). Also, it has been thought that elevated prostaglandin E2 (PGE_2_) may contribute to nociceptive behavior mediated by TRPV4 (Transient receptor potential cation channel subfamily V member 4) which is known to be associated with inflammatory and neuropathic pain via p38 MAPK pathway (48). As the examples written here indicate, PUFA metabolites play critical roles in intracellular molecular signalizations, especially where inflammation (during both initiation, progression, and solution phases) is at the center (**Figure 2**).

## 3 Inflammasome complexes, the center of the cellular inflammatory response

To the broadest extent, inflammation is a protective response to both exogenous factors, including physical (e.g. UV), chemical (e.g. hydrogen peroxide), or biological (pathogens) and endogenous signals such as damaged cells to result in the elimination of the reason for injury and tissue repair (49). Together with the complex molecular, immunological, and physiological processes of the inflammatory response (49), inflammasome protein complexes of the innate immune system stand as the central molecules activating inflammatory responses (50). Thus, they are defined as new potential targets for novel anti-inflammatory drug development (50). The functionality and efficiency of inflammatory complexes are crucial not only for the protection of organisms against pathogens but also for mediating control over sterile insults (51). Aberrant inflammasome signaling appears in the development and worsening of different diseases (cardiovascular and metabolic diseases, cancer, and neurodegenerative disorders) accompanied by oxidative stress and associated chronic inflammation (51).

**Figure 3:**
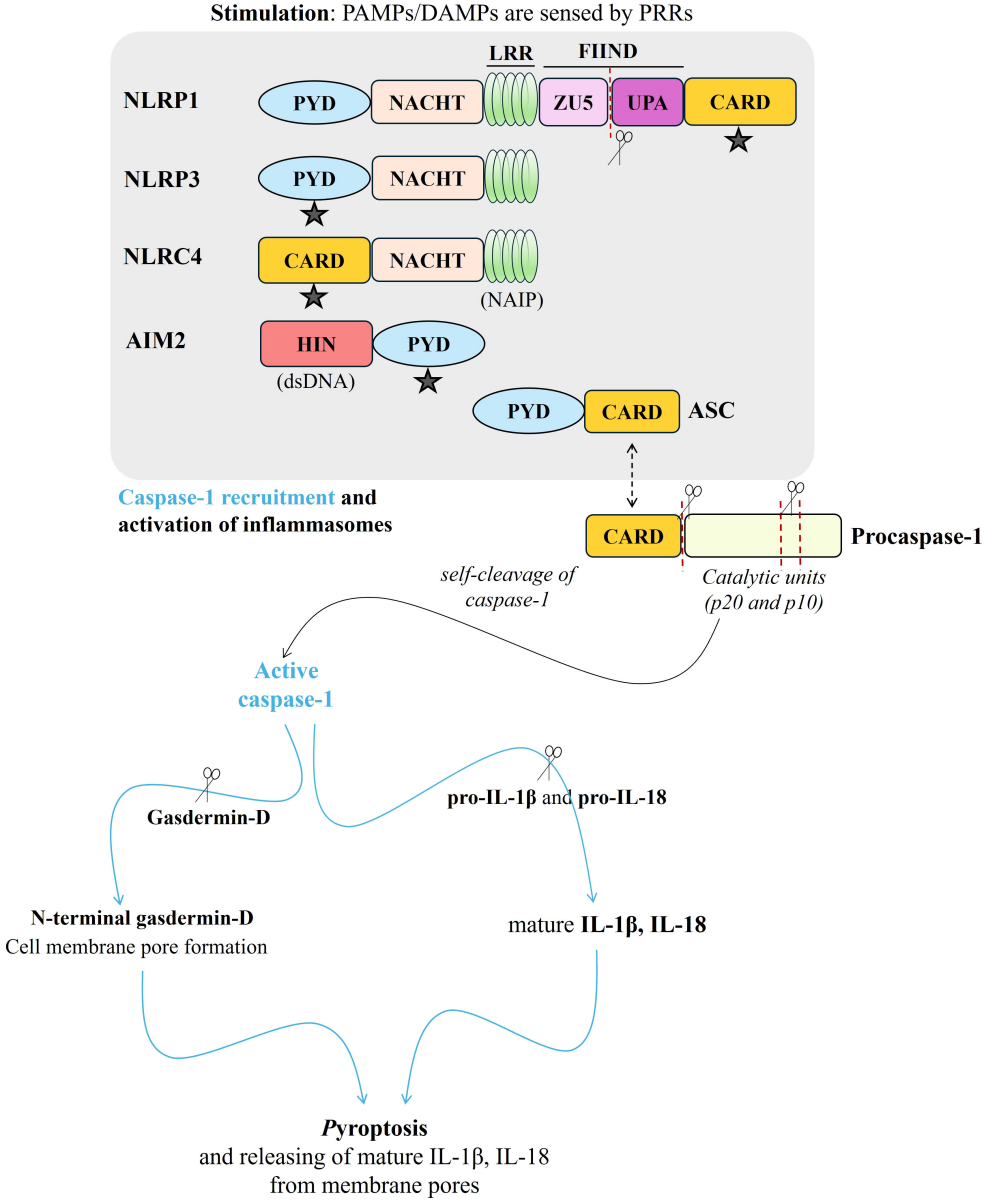
A brief scheme presenting structure and activation of NLRP1, NLRP3, NLRC4, and AIM2 inflammasomes via recruitment of ASC (“
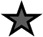
” indicates ASC binding site through the CARD-CARD or PYD-PYD interaction), and then, procaspase-1 resulting in activation of mature caspase-1. Caspase-1 activation causes the cleavage of pro- IL‐1β, pro-IL‐18, as well as gasdermin-D. N-terminal gasdermin-D promotes the perforation of the cell membrane and mature IL‐1β and IL‐18 release from the membrane pores, meantime, pyroptosis - inflammatory cell death - appears. (red-dotted lines and “
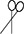
“were used for cleaving. PAMPs, pathogen-associated molecular patterns; DAMPs, damage-associated molecular patterns; PRRs, pattern recognition receptors; PYD, pyrin domain; NACHT, a central nucleotide-binding and oligomerization domain (NOD); LRR, leucine-rich repeats; FIIND, function-to-find domain containing ZU5 and UPA domains; CARD, caspase recruitment domain; NAIP, NLR family apoptosis inhibitory protein; HIN, hematopoietic, interferon-inducible, and nuclear localization; dsDNA, double-stranded DNA; ASC, apoptosis-associated speck-like protein containing CARD; Procaspase-1, immature caspase 1; Pro-IL-18, immature interleukin 18; Pro-IL-1β, immature interleukin 1β; IL‐18, interleukin 18; IL‐1β, interleukin 1β).

Concerning the human immune system, pathogen-associated molecular patterns (PAMPs) or damage-associated molecular patterns (DAMPs), are sensed by the pattern recognition receptors (PRRs) [Toll-Like Receptors (TLRs), Nucleotide-binding oligomerization domain-Like Receptors (NLRs), RIG-I-Like Receptors (RLRs), C-type Lectin Receptors (CLRs)]. Following this stimulation, inflammasomes are formed, and associated downstream signals are promoted (52, 53). The well-characterized inflammasomes are classified as NLRP1 (NLR family pyrin domain-containing protein 1), NLRP3 (NLR family pyrin domain-containing protein 3), >NLRC4 (NLR family CARD domain-containing protein 4), and AIM2 (absent in melanoma 2) (**Figure 3**). The formed multiprotein complex can be completed with a caspase effector (caspase-1), as well as adapter protein ASC (apoptosis-associated speck-like protein containing CARD) (54). In general, upon a signal indicating infection or cellular damage by other receptor stimuli, NLR (nucleotide-binding domain, leucine-rich repeat containing) or PYHIN family (in case of AIM2) oligomerizes and a larger multiprotein complex is formed by recruiting additional components (54). Procaspase-1 is included via ASC recruitment and then undergoes autoproteolytic cleavage, and active mature caspase-1 is released to induce processing and secretion of mature IL‐1β and IL‐18 (55). In parallel, pyroptosis, inflammatory cell death, appears due to the cleaving of gasdermin D (GSDMD) by mature (active) caspase-1 and the cell membrane is perforated – where mature IL‐1β and IL‐18 release – by the N-terminal GSDMD (**Figure 3**) (55).

NLRP1, the first identified inflammasome, is widely expressed in various types of cells with a majority in immune and epithelial cells. It is associated with the development of autoinflammatory diseases and cancers (56, 57). NLRP1 is described as the main skin inflammasome, and its importance is also mentioned concerning inflammatory skin diseases such as psoriasis (58, 59). Moreover, attention is drawn to the close relationship between NLRP1 dysfunction and carcinogenesis, including the development of skin cancers, e.g. melanoma (60). The full isoform-1 structure of NLRP1 consists of N-terminal pyrin domain (PYD), NACHT domain, a central nucleotide-binding and oligomerization domain (NOD), NLRC4 helical domain, C-terminal leucine-rich repeats (LRRs), FIIND domain (function-to-find domain, ZU5-UPA domain), and C-terminal caspase recruitment domain (CARD) (54). It has been shown that autolytic proteolysis of NLRP1 is promoted by autolytic cleavage at Ser^1213^ within the FIIND domain, and in the next step, ASC recruitment to the C-terminal CARD domain of the processed NLRP1 molecule (61). Autoproteolysis occurs when the N-terminal fragment is degraded and the UPA-CARD domain is released (62). Both the FIIND and CARD domains of NLRP1 are essential for its activity. Moreover, PYD and LRR domains were associated with a potential for self-inhibition function (63). Together with all, the potential function of the PYD domain of human NLRP1 (missing in murine NLRP1) remains still an important point for research.

Furthermore, it has been indicated that small-molecule inhibitors (such as Val-boroPro as known as PT-100 or Talabostat) of dipeptidyl peptidases 8 and 9 (DPP8/DPP9) can interact with NLRP1 and activate it (64). Even, DPP9 is suggested as a checkpoint for NLRP1 inflammasome activity by showing quenching activity of DPP9 for low levels of NLRP1 C-terminal (64). Moreover, ubiquitous endogenous TRX-mediated suppression of the NLRP1 activity (via redox-active cysteines, Cys^32^ and Cys^35^, of TRX in NLRP1 binding) has been indicated (65). However, no binding interaction was found between thioredoxin-1 (TRX1) and CARD8 (a caspase-1-activating inflammasome having just FIIND domain, and CARD domain, different than NLRP1), suggesting oxidized TRX1 may reduce NLRP1-TRX1 interaction which is an activation signal (66–68). In addition, the literature showed that protein folding stress can promote NLRP1 and CARD8 activation induced by DPP8/9-inhibitors (including Val-boroPro) via accelerating NLRP1 and CARD8 N-terminal degradation (69).

In contrast, NLRP3 can be activated by diverse molecular and cellular signals coming from microbial infection and cellular damage including ionic flux, mitochondrial dysfunction, excessive ROS generation, and lysosomal damage (70). It plays a critical role in inflammatory macrophage activation and regulatory T-cell differentiation (71, 72). Impaired signaling in the NLRP3 pathway has been shown associated with various autoimmune and metabolic disorders such as type 2 diabetes, Alzheimer’s disease, and cardiovascular diseases (73). NLRP3 structure consists of NLRP3 domain (PYD, NACHT, and, LRR), adaptor protein ASC (PYD and CARD), and an effector full-length caspase-1 (74). NLRP3 inflammasome activation involves two steps, the priming and activation steps. The priming step (regarding canonical NLRP3 pathway) involves NF-κB-promoted up-regulation of NLRP3, pro-IL-1β and pro-caspase-1 expression mediated by TLR-adaptor molecules myeloid differentiation primary response 88 (MyD88) (75), and NLRP3 deubiquitination (51). Priming also includes different post-translational modifications (PTMs) of NLRP3, ASC, and caspase-1 accompanied by metabolic changes from oxidative phosphorylation to glycolysis (76). Activation stimuli are accompanied by NLRP3 re-localization to mitochondria together with mitochondrial ROS, mitochondrial DNA, or cardiolipin releasing into the cytosol, potassium efflux through ion channels, and cathepsins release following destabilization of lysosomal membranes (77). Without any activation stimuli, an inactive conformation is observed due to the NACHT domain, having ATPase activity, and binding to the LRRs and/or the PYD (76). Regarding the activation, NACHT subdomains are conformationally re-arranged induced by ATP hydrolysis and interacted with mitotic serine/threonine kinase NEK7 (78), mediating the establishment of bridges adjacent to NLRP3 subunits with bipartite interactions at the oligomerization interface (79). Then, the ASC adaptor protein is recruited into the inflammasome structure and subsequent caspase-1 recruitment is completed (76).

In addition to the canonical activation of NLRP3 (TLR4 stimulation by interacting with the outer lipopolysaccharide (LPS) membrane of gram-negative bacteria, as mentioned above), NLRP3 can be also activated in a non-canonical way (80). Independently of TLR4 activation, murine pro-caspase-11 and its human orthologs pro-caspases-4 and -5 can directly bind intracellular LPS, and promote NLRP3 non-canonical activation as well as IL-1β, IL-18 release, and pyroptosis (75, 77). Regardless of the literature data showing mouse caspase-11-initiated NLRP3 and ASC-dependent activation of caspase-1, there is still a gap in the information regarding potential inflammasome-associated proteins (other than guanylate-binding proteins, GBPs) in the LPS sensing of caspase-4 as well as the molecular contribution of caspase-1, caspase-4 and NLRP3 (81).

Activation of NLR family apoptosis inhibitory protein (NAIP)-NLRC4 inflammasome is generally centered on mounting an immune response against gram-negative bacteria, such as *Salmonella Typhimurium*, but together with that, recent findings also show the role of this inflammasome in autoinflammatory diseases (82) as well as in cancers such as glioma and breast cancer (83). NLRC4 consists of an N-terminal CARD domain, a central NACHT domain, and a C-terminal LRR domain (82). Following a trigger signal such as cytosolic flagellin, NLRC4 can directly recruit pro-caspase-1 via CARD-CARD interaction as well as indirectly with ASC adaptor protein-CARD interaction and trigger downstream caspase-1 processing activation (82). It has been shown that human NAIP protein, can sense type III secretion system pathogen components and promote activation of the NLRC4 via conformational change (84). Also, NLRC4 expression has been known to be upregulated by pro-inflammatory stimuli such as TNFα as well as through genotoxic stress-mediated p53 activation (85).

In defense of the host not only against external pathogens (both viruses and bacteria) but also against the development of inflammatory and autoimmune diseases (e.g. psoriasis) and cancer, the activity of the AIM2 inflammasome plays an important role (86). It consists of 2 domains: the N-terminal PYD domain and the C-terminal HIN (hematopoietic, interferon-inducible, and nuclear localization) domain (86). AIM2 can bind double-stranded DNA (dsDNA) via its HIN domain which will initiate oligomerization by recruiting ASC containing CARD domain that will turn procaspase-1 recruitment (87). Also, AIM2 binds to ASC via PYD-PYD interaction (51). This inflammasome can be activated by both host and pathogen antigens (dsDNA delivered from bacteria, DNA viruses) (88). Moreover, it was found that the AIM2 inflammasome is activated by neutrophil extracellular traps (NETs), SARS-CoV-2, or influenza A, as well as by mitochondrial damage. Like NLRP3, it can induce both apoptosis (via activation of caspase-8) and pyroptosis (via activation of caspase-1) and secretion of IL-1β and IL-18 (87). Moreover, it was suggested a “DNA dose-dependent switch between apoptosis and pyroptosis” due to the parallel activation of NLRP3 and AIM2 (87). The importance of regulation of the AIM2 inflammatory response has been indicated in activation, intensity, and duration levels to optimize inflammation (89).

It is known that fragmentation/cyclization products of PUFAs (e.g. 4-HNE, MDA, 8-isoprostanes), the generation of which increases significantly under the influence of oxidative stress, cause several biological consequences by affecting the antioxidant system, inflammation, as well as apoptosis through the formation of protein adducts (90). These adducts’ formation modifies protein structure and affects their functions, and ultimately, molecular pathways, in which these proteins participate (91). For instance, 4-HNE is found to inhibit the NF-κB pathway and lead the upstream kinase IKK (IκB kinase) for inactivation of anti-apoptotic B-cell lymphoma-2 (Bcl-2) and alter cell survival status (92). The serum level of 8-isoprostanes (8-iso-PGF2α) is increased as a rapid response during the acute phases of the inflammatory response, resulting in crosstalk with cytokines to activate NF-κB and subsequent gene expression of pro-inflammatory cytokines as well as more COXs, in case of chronic inflammation (93).

**Figure 4:**
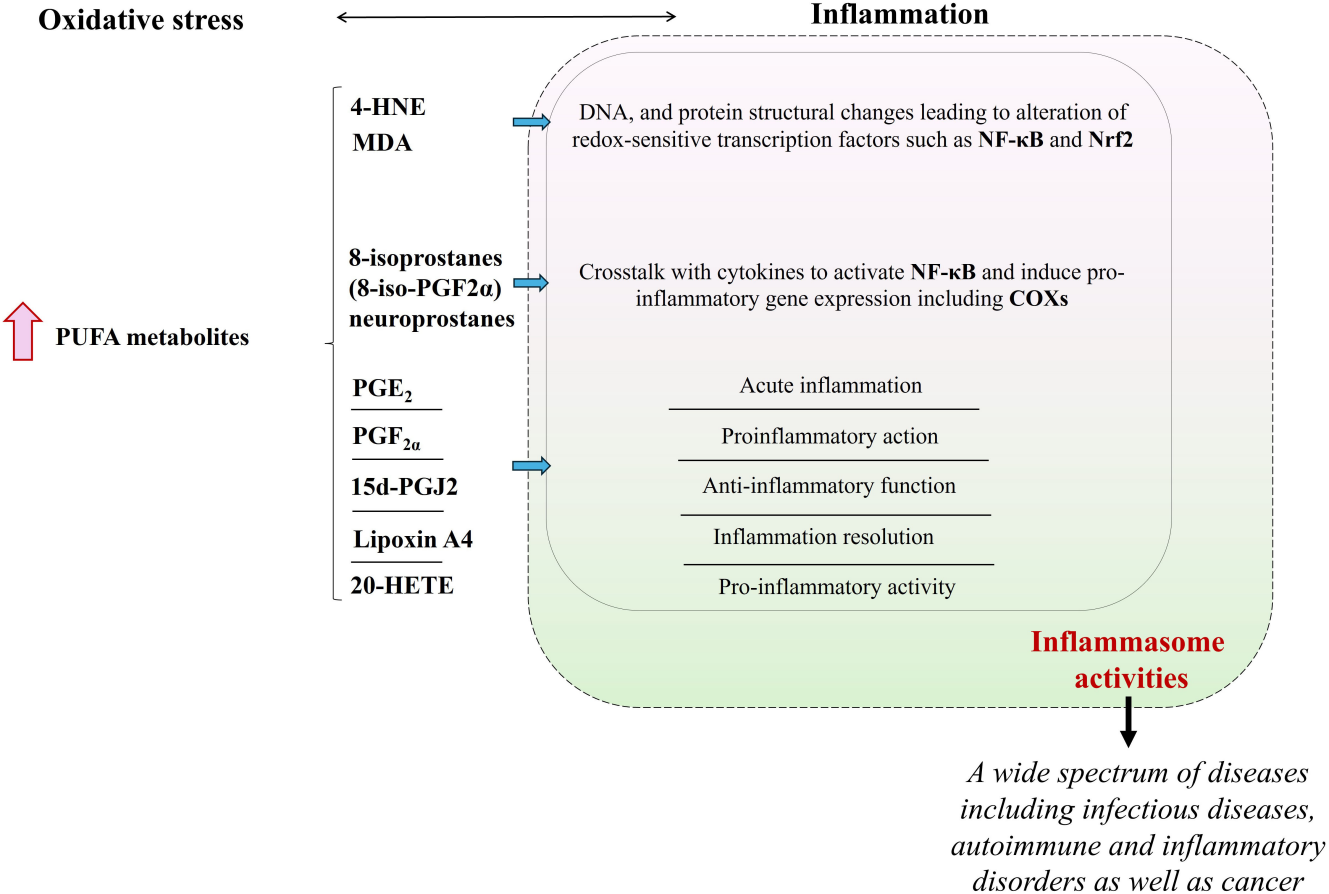
The consequence of an excessive increase in the level of ROS/RNS as a result of oxidative stress is the increased generation of PUFA metabolites that influence the cellular inflammatory response in which the activity of inflammasomes plays a key role. PUFA metabolites that are found to be prominent in the regulation of inflammation are shown in the figure. (“
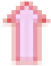
” is used for elevation. PUFA, polyunsaturated fatty acid; 4-HNE, 4-hydroxy-2-nonenal; MDA, malondialdehyde; NF-κB, nuclear factor - kappa B; Nrf2, Nrf2, nuclear factor 2 associated with erythroid 2; 8-iso-PGF2α, 8-iso prostaglandin F 2α; COXs, cyclooxygenases; PGE_2_, prostaglandin E2; PGF_2a_, prostaglandin F2α; 15d-PGJ2, 15-deoxy-∆-12,14-prostaglandin J2; 20-HETE, 20-hydroxyeicosatetraenoic).

Moreover, eicosanoids play key roles in every stage of inflammation, as well as in apoptosis (46). Some examples follow: inflammation resolution by lipoxin A4 (94), the pro-inflammatory activity of 20-hydroxyeicosatetraenoic (20-HETE) through NF-κB stimulation (95), inhibition of apoptosis by 15-HETE which strengthened phospho-Akt and heat shock protein 90 (96), acute inflammation induced by prostaglandin E2 (PGE_2_) through mast cell activation via the EP3 receptor (97), proinflammatory action of prostaglandin F2α (PGF_2α_) by increasing the level of pentraxin-3 (98) and, anti-inflammatory functions of 15-deoxy-∆-^12,14^-prostaglandin J2 (15d-PGJ2) (99). Therefore, altered lipid metabolism caused by oxidative stress, manifested by the generation of PUFA metabolites presents a great potential for a direct/indirect effect in the modulation of inflammasome activities that are standing at the center of inflammation (**Figure 4**). As a matter of fact, it has been revealed that lipid metabolism significantly participates in the modulation of inflammation in the context of acute and chronic diseases (3).

## 4 The influence of PUFA metabolites on the activity of inflammasomes

Lipid metabolism, participating in maintaining cellular homeostasis, is highlighted for regulation of inflammasome activation, as lipid remodeling is critically important for several diseases, including cardiovascular and metabolic diseases as well as cancer (**Figure 4**) (100, 101). Therefore, the biological implications of the effect of PUFA metabolites on inflammatory activities offer a new perspective for the development of therapeutic approaches.

In this respect, the importance of the regulatory effect of PUFA metabolites on inflammasome activities has been highlighted in both *in vitro* and *in vivo* studies. Anti-inflammatory effects of 15-LOX metabolites of α-linolenic acid have been shown in LPS-induced inflammation in both mouse macrophage cell line RAW 264.7 and peritoneal macrophages isolated from BALB/c male mice, which was mediated by inactivating NLRP3 inflammasome and downregulation of caspase-1 (102). In mouse peritonitis models of gout and murine anthrax infection, 15d-PGJ2 inhibited caspase-1 activation by the NLRP1 and NLRP3 inflammasomes (103). It has also been demonstrated in both bone-marrow-derived macrophages and phorbol myristate acetate-differentiated human monocytic leukemia cells that LXA4 can inhibit NLRP3 inflammasome formation by suppressing oxidative stress at the upstream of NLRP3 activation (through dropping NADPH oxidase activation, ROS generation, and mitochondrial dysfunction as well as modulating Nrf2 activity) (104). Below, we will analyze the interference of PUFA metabolites in inflammasome activities mentioned here, focusing on NLRP1, NLRP3, AIM2, and NLRC4.

### 4.1 The NLRP1 inflammasome

**Figure 5:**
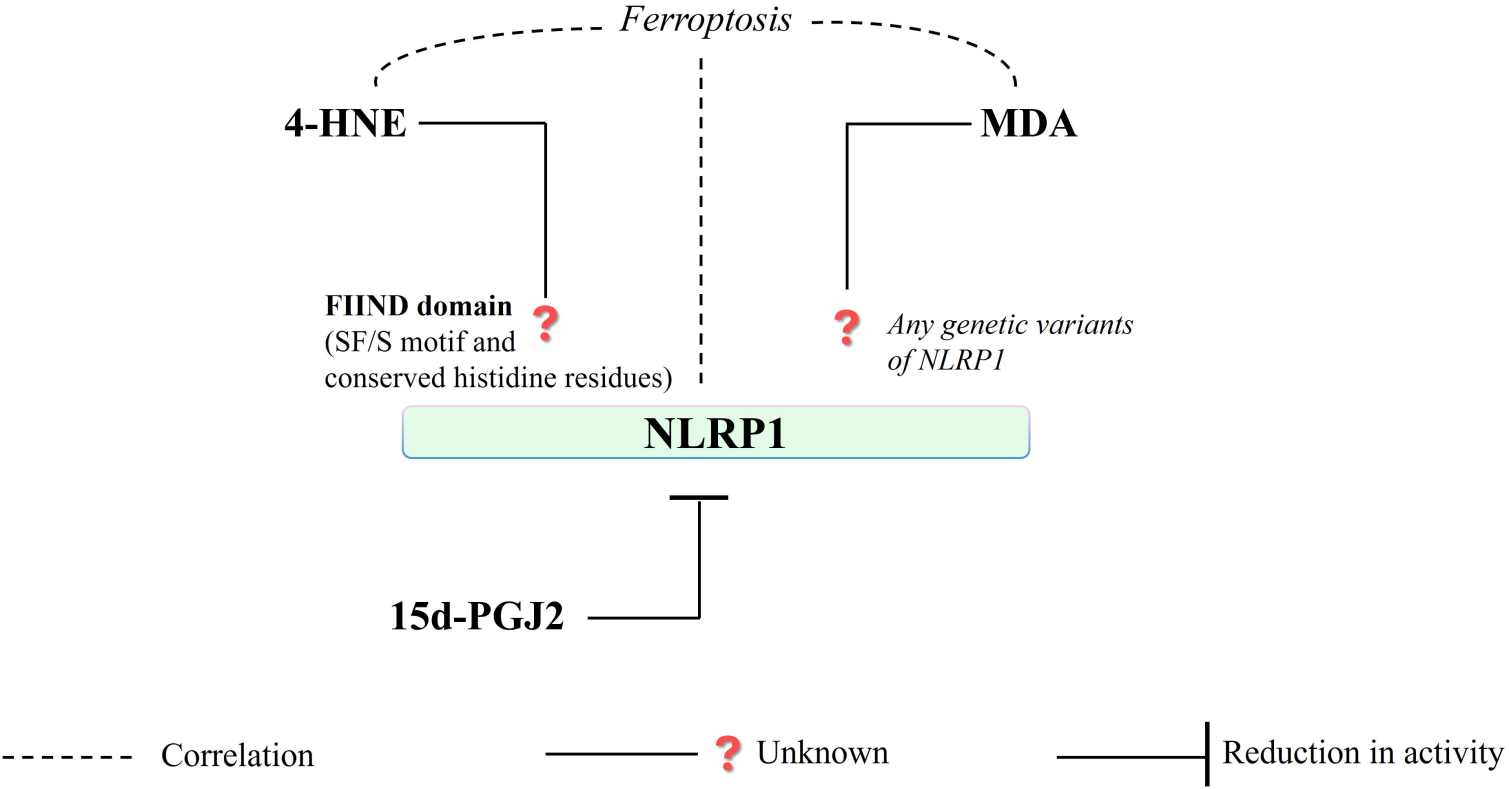
Regarding redox-sensitive amino acids (SF/S motif, conserved histidine residues) in FIIND domain structure, NLRP1 is a protein presenting a high potential for the formation of 4-HNE - protein adducts that are mainly connected to ferroptosis. Consequently, MDA is unlikely to affect protein structure or related gene expression (in the case of NLRP1). (4-HNE, 4-hydroxy-2-nonenal; MDA, malondialdehyde; NLRP1, NLR family pyrin domain-containing protein 1; 15d-PGJ2, 15-deoxy-∆-12,14-prostaglandin J2; S, serine; F, phenylalanine).

NLRP1 is the major inflammasome sensor in human skin and an important component of the cellular proinflammatory response regarding the innate immune system. As we mentioned above, although it was the first identified inflammasome, there are still many questions arising from its structure and associated activation as well as self-inhibition. At this point, the regulatory effect of the lipid metabolites on this protein complex may be critical from the point of its functionality. The FIIND domain of NLRP1 consists of SF/S motif, conserved histidine residues, amino acids susceptible to oxidation (105–107) which indicates a great potential and efficiency of post-translational autocleavage (54). Therefore, assessing the role of potential oxidative modifications (e.g. via the formation of protein adducts with 4-HNE – Michael adduction (106, 108)) in terms of redox-sensitive amino acids may be also important for NLRP1 activation, especially regarding the FIIND domain (**Figure 5**). Even, a recent study in human keratinocytes exposed to ozone (O_3_) showed that NLRP1 is a protein that forms adducts with 4-HNE, which may result in its proteasomal degradation and/or activation via E3 ubiquitin ligase UBR2 (109).

Moreover, another study focusing on the interaction between inflammasome complexes and ferroptosis, using an H_2_O_2_-mediated oxidative stress model in HTR-8/SVneo placental trophoblast cells, showed a decreased level of intracellular MDA accumulation and cell death, together with the increased glutathione peroxidase 4 (GPX4) expression and increased glutathione (GSH) level, resulting from NLRP1 silencing (110). The same study also demonstrated a positive correlation between NLRP1, NLRP3, IL-1β, and caspase-1 expression levels and ferroptosis in the human trophoblast cell line (110). However, other work has suggested that the formation of NLRP1, as well as NLRP3, might be a critical step for ferroptosis (65). In this situation, as a result of intensive oxidation of phospholipid PUFAs, lipid hydroperoxides accumulate, which is accompanied by an increase in the generation of 4-HNE and MDA, and consequently, the permeability of the cell membrane increases and it may even lead to rupture of the cell membrane (111). Therefore, iron accumulation and lipid peroxidation appear to be two critical elements promoting oxidative damage of the membrane during ferroptosis (112). Moreover, ferroptosis and pyroptosis may act synergistically in the cellular immune response (112). Therefore, the potential formation of 4-HNE adducts of NLRP1 may be also analyzed in detail regarding the relationship between inflammasomes and ferroptosis mentioned above. Depending on the intensity of oxidative stress, reflected in the generation of 4-HNE, NLRP1 activity may be regulated both directly by NLRP1 structural changes resulting from the formation of adducts with 4-HNE and by other intracellular changes that result from modifications in the ROS level and ion balance - regarding NLRP1 upstream signaling. This may induce synergistic ferroptosis and pyroptosis reactions, modulating NLRP1 activity. The role of the 4-HNE-protein adducts in sensitizing cells to ferroptosis, inducing ferroptosis, as well as cell resistance to ferroptosis has also been indicated (113). Although 4-HNE is mainly attributed to protein adduct formation, especially regarding ferroptosis (113), no direct interaction with NLRP1 has been demonstrated so far (114). It has been indicated that such protein modification can lead to protein instability and unfolding, by accessing protein folds or binding pockets (91), and even, protein cross-linking and aggregation due to the decreased efficiency of proteasomal degradation (115).

However, for innate immune response, eicosanoids, with anti- or pro-inflammatory activity, are critical, as mentioned above. Literature data regarding eicosanoids associated with NLRP1 activity is very limited. It was shown that anti-inflammatory 15d-PGJ2, a peroxisome proliferator receptor-γ (PPAR-γ) agonist, was able to prevent the autoproteolytic activation of caspase-1 and the maturation of IL-1β, probably by affecting in *de novo* protein biosynthesis, rather than its direct modification of caspase-1, as suggested by the authors (103). Moreover, it was demonstrated that 15d-PGJ2 can inhibit lethal toxin-mediated NLRP1 activity (**Figure 5**) in a murine infection model (103). Here, a part of the direct effect, the anti-inflammatory and antioxidant role of 15d-PGJ2 may be assessable in the regulation of NLRP1 activity by enhancing PPAR-γ transcriptional activity, inhibiting the NF-κB, and JAK-STAT pathways (116).

This is why NLRP1 and its genetic variants are key therapeutic targets in a wide variety of autoimmune and inflammatory disorders, mainly in inflammatory skin diseases (62). Both the changes in protein structures affected by interaction with PUFA metabolites and its functional consequences, as well as the single nucleotide polymorphism in the *Nlrp1* gene indicated by recent studies, opens a promising field to develop targeted therapies against diseases in which NLRP1-associated inflammation is centered (58).

### 4.2 The NLRP3 inflammasome

The NLRP3, due to its various stimulation network including both external and internal causes mentioned above, is a critical component of the innate immune system (70). In addition, any other functions in the cytoplasm or nucleus, as well as mechanisms relating to non-canonical NLRP3 activity, remain of interest (117). Considering the critical participation of endoplasmic reticulum (ER) stress and mitochondrial dysfunction in the NLRP3 stimulation, the roles of the reactive aldehydes and enzymatically generated metabolites of PUFAs appear as much more important regarding structural changes and alterations in protein-protein interactions.

First of all, it was found that as a result of the activation of aldehyde dehydrogenase 2 (ALDH2), which protects mitochondria through the metabolism of toxic aldehydes such as 4-HNE and MDA, the activation of the NLRP3 inflammasome is weakened and, consequently, pyroptosis is inhibited (118). However, the molecular mechanism resulting from this activity of ALDH2 has not been clearly explained. It has been suggested that NLRP3-activated upstream ROS regulation may play an important role in the regulation of NLRP3 by ALDH2 (118). Furthermore, a study on RAW264.7 cells showed that Alda-1, an ALDH2 activator, was able to inhibit both phases of NLRP3 (119). Thus, the reduction in NLRP3 activity may be explained through the cytoprotective activity of ALDH2 by reducing ROS generation and inflammation accompanied by a reduction in 4-HNE level, p65 (RelA), and p38 activation (120).

It is known that the Keap1-Nrf2 interaction is disrupted as a result of the formation of 4-HNE-Keap1 adducts, which prevents Nrf2 degradation and increases Nrf2 signaling with an enhanced antioxidant response, and NF-κB activation is blocked by inhibition of IKK activity through interaction with 4-HNE (121). Moreover, the action of 4-HNE at the physiological level (3 µM) on human and mouse macrophages showed that, independently from Nrf2 and NF-κB signaling, 4-HNE through direct interaction with NLRP3 and inhibition of its interaction with NEK7, acts as an endogenous inhibitor activation of the NLRP3 inflammasome and associated inflammation (121). Another study demonstrated that 4-HNE (at 30 µM) - induces NLRP3 at mRNA level, NLRP3 activity, and generation of IL-1β in the human retinal pigment epithelial cell line (122). Thus, the effect of 4-HNE on the NLRP3 activity may depend on the severity of oxidative stress, and by taking into account the differences in the cellular micro-environment that come from cell specificity. Additionally, it is suggested that another type of lipid peroxidation products - cyclic 8-isoprostanes - may be also related to NLRP3 activity and associated IL-8 generation, the high concentration of which in blood serum was also found to be correlated with severe liver fibrosis (123). It is also possible to link the action of 8-isoprostanes, as one of the biomarkers of oxidative stress (124), with mitochondrial dysfunction (125) and oxidative modifications of ER proteins resulting from stress on the endoplasmic reticulum, which is considered to be dependent on NLRP3 activation (126, 127).

**Figure 6:**
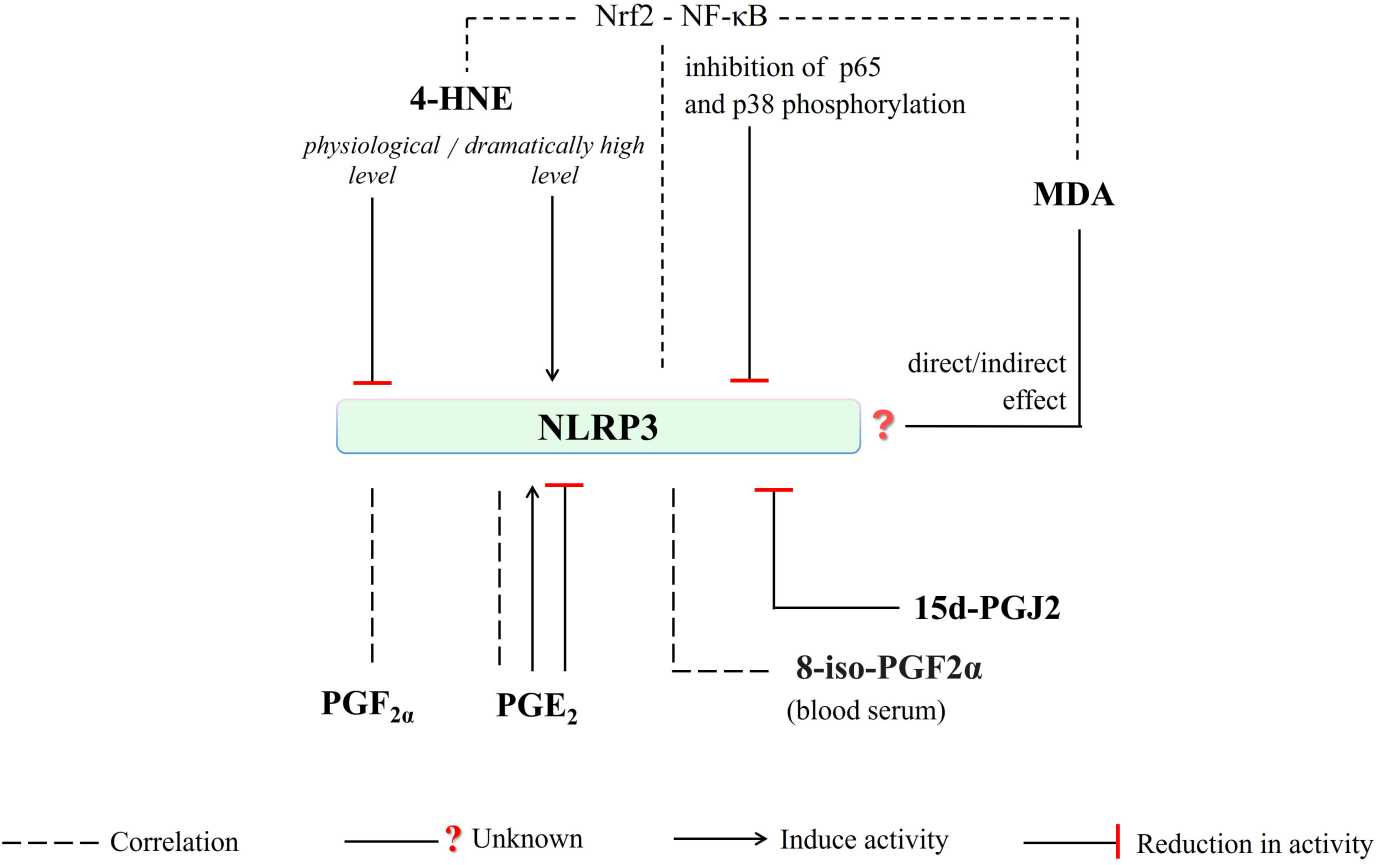
4-HNE, depending on the intensity of oxidative stress, may directly or indirectly (via interfering Nrf2-NF-κB crosstalk) induce or reduce NLRP3 activity. Any direct/indirect effect of MDA has been not identified yet. The generation of PGF_2a_, PGE_2,_ and 8-iso-PGF2a (blood serum level) is correlated with NLRP3 activity. Moreover, PGE_2_-mediated induce or reduce in NLRP3 activity has been reported. And, NLRP3-mediated caspase-1 activation is inhibited by 15d-PGJ2. (4-HNE, 4-hydroxy-2-nonenal; MDA, malondialdehyde; NLRP3, NLR family pyrin domain-containing protein 3; 15d-PGJ2, 15-deoxy-∆-12,14-prostaglandin J2; PGE_2_, prostaglandin E2; PGF_2a_, prostaglandin F2α; 8-iso-PGF2α; 8-iso Prostaglandin F 2α; p38, a member of mitogen-activated protein kinases (MAPKs); p65, RelA, a heterodimer in the NF-κB signaling pathway).

Moreover, the differences in the action of inflammation-inducing factors and even epigenetic variabilities, considering the link between oxidative stress, lipid metabolism, and epigenetics, may affect this situation (128, 129). A study assessing the effects of palmatine (PAL), a natural isoquinoline alkaloid, showed an increased antioxidant response (enhanced superoxide dismutase, SOD, and GSH levels) by reducing MDA production in THP-1 macrophages and by reducing p65 (RelA) and IκBα (inhibitory κB-α) phosphorylation and inhibition of NLRP3 (130). However, a study on rats showed a reduction in IL-6 and tumor necrosis factor-alpha (TNF-α) levels, both at the protein and mRNA levels, as well as inhibition of p65 and p38 phosphorylation, while reducing MDA and increasing GSH levels as a result of cyclophosphamide treatment (131). Moreover, oxidative stress causing excessive MDA production may promote a p65-mediated inflammatory response (132). An *in vivo* study in an oral mucositis model highlighted flavocoxide-mediated inhibition of NLRP3 activity and its downstream signals (caspase-1, IL-1β, and IL-18) by reducing NF-κB expression as well as MDA production (133). Aldehydes, which are products of lipid peroxidation, can alter proteins and DNA structure, and transduce activation of NF-κB signaling pathways in a wide spectrum of molecular changes involving alterations of kinase and transcription factors activity as well as DNA and protein damage (134). Although a direct relationship between NLRP3 components and MDA has not been demonstrated, by regulating NF-κB activity, MDA may also influence the NLRP3 initiation process. Depending on the severity of oxidative stress, 4-HNE, and MDA have the potential to be game changers in the NLRP3-mediated inflammatory response, either directly or indirectly (**Figure 6**).

However, NLRP3 activation in innate immune cells is accompanied by the generation of other lipid mediators, such as pro-inflammatory eicosanoids (135). Studies conducted on fasted and fed mice (C57BL/6) showed that increased AA generation in fasting condition resulted in inhibition of the NLRP3-dependent generation of both IL-1β and IL-18 via blocking phospholipase C and further activities of protein kinases PKD and JNK (136). Furthermore, COXs inhibition was able to reduce eicosanoid levels but did not affect arachidonic acid (AA) level. Decreased generation of PGE_2_ and PGF2α was also demonstrated along with a decrease in IL-1β levels in bone marrow-derived macrophages from LPS-initiated mice with nigericin (136). However, a decrease in free AA and diminished PGE_2_ secretion due to gamma-tocotrienol (γT3) - an unsaturated vitamin E - treatment was indicated in the differentiated bone marrow-derived macrophages obtained from C57BL/6 mice (135). PGE_2_-stimulated IL-1β production in response to *Tytius serralatus* venom via protein kinase A (PKA) activation was also shown (137). Furthermore, a study on human primary monocyte-derived macrophages showed that NLRP3 inflammasome activation was inhibited by PGE_2_ through stimulation of PGE_2_ receptor subtype 4 (EP4) and an increase in intracellular cAMP (138). It is important to mention that the variety of the eicosanoid production tendency and its effects on NLRP3 activity (**Figure 6**) may be dependent on the intensity of the cellular inflammatory response, correlated with oxidative stress, regarding concentration dependency as suggested by the authors (136).

It has been pointed out that activation of NF-κB induced by TLR/CD40 engagement on dendritic cells can induce IL-23 p19 gene expression, it can also promote dendritic cells to induce COX2 expression and PGE_2_ generation, which amplifies NF-κB signaling through the EP2/EP4-cAMP-PKA-CREB pathways (139). Together with that, macrophage COX‐2 and PGE_2_ levels are increased by NF-κB activity (activated by TNF‐α and LPS), which turn EP2 receptors improving NF‐κB signaling. This increases the recruitment of macrophages to inflamed site and promotes chronic inflammation (139). Moreover, a study in rat alveolar macrophages showed that TNF-α can also be suppressed by PGE_2_ (synthesis via NO production in response to LPS) through protein kinase A anchoring proteins implicated PKA regulatory subunit type II (RII) (140). Thus, the modulatory effect of PGE_2_ on NF-κB signaling may be a key point for NLRP3 activity, as a feedback mechanism, depending on cell type and oxidative stress severity.

Additionally, it is known that cyclopentenone prostaglandins (cyPGs) including PGA2, PGA1, and PGJ2 and their metabolites may be involved in regulating the inflammatory response by interfering with the NF-κB, AP-1, MAPK, and JAK/STAT signaling pathways (141). Moreover, 15d-PGJ2 may inhibit NF-κB gene expression by covalently modifying cysteine ​​residues of IκB kinase and DNA-binding domains of NF-κB subunits (142). The mechanism includes cyPG-mediated modification of cysteine ​​179 in the activation loop of the IKKβ subunit and inhibition of its phosphorylation (141). Moreover, 15d-PGJ2 was also found to inhibit NLRP3-mediated caspase-1 activation (103). Similar to the negative regulation of NLRP1 activity, there may be upstream regulation of NLRP3 activity through the inhibition of NF-κB by 15d-PGJ2. NLRP3 activity is critical for maintaining cellular homeostasis and physiological metabolism (143). And, the impairment of its activity is important for a wide spectrum of diseases including infectious diseases, cancer, atherosclerosis, diabetes, and obesity (143). Therefore, analyzing the effect of PUFA metabolites interfering with both the priming and activation stages of NLRP3 may bring opportunities for developing new therapeutic approaches. Moreover, it may offer a new perspective regarding its non-canonical activation, where currently our knowledge is still limited.

### 4.3 The NAIP–NLRC4 inflammasome

**Figure 7:**
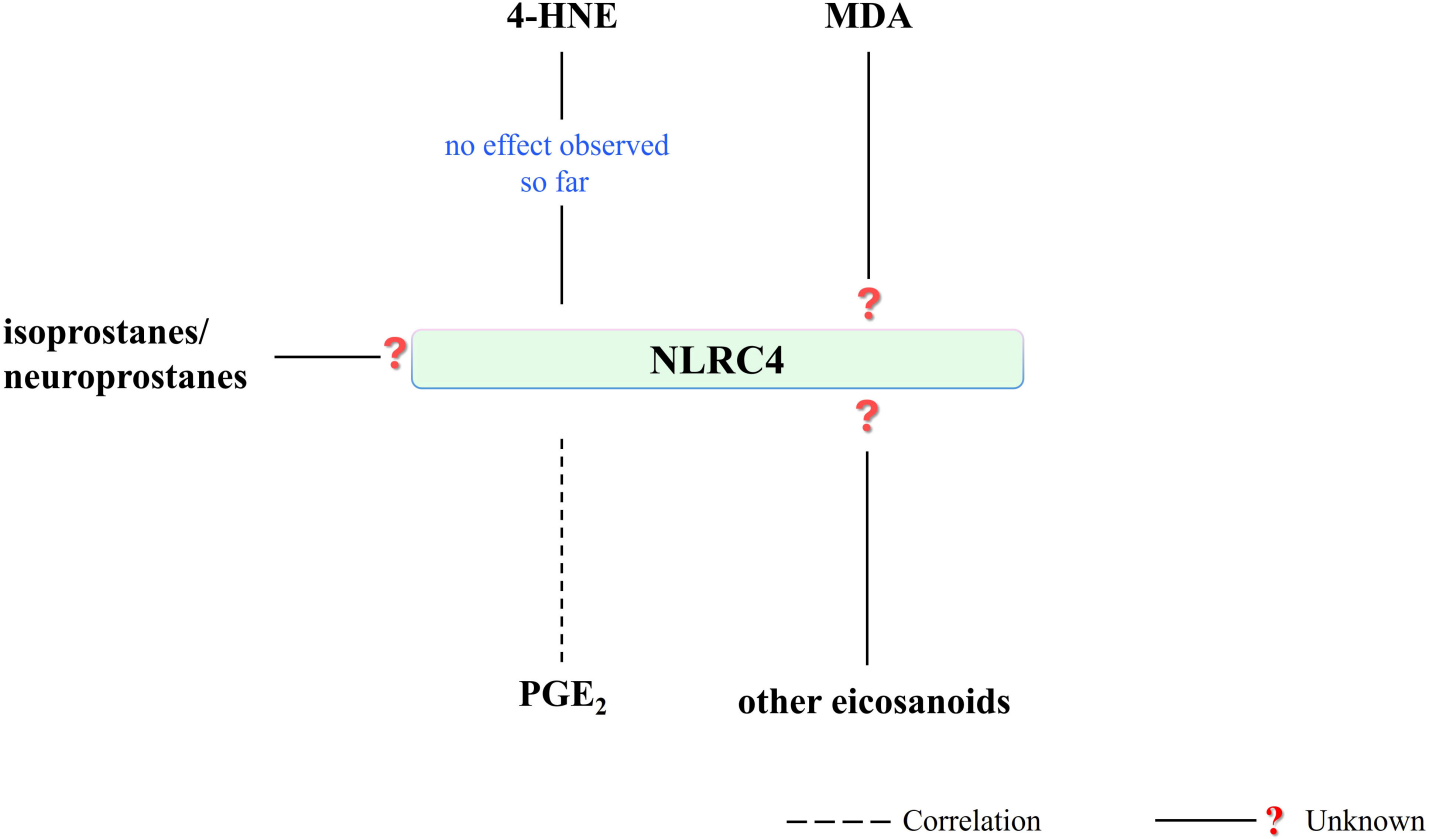
Literature on the effect of PUFA metabolites on NLRC4 activity is very limited. 4-HNE has presented no effect on NLRC4 activity and associated IL-1β release so far. NLRC4-mediated PGE_2_ production has been shown. On the other hand, there is a research need for the effect of other eicosanoids or PUFA metabolites on NLRC4 activity. (4-HNE, 4-hydroxy-2-nonenal; MDA, malondialdehyde; NLRC4, NLR family CARD domain-containing protein 4; PGE_2_, prostaglandin E2).

NLRC4 is a key inflammasome in an immune response to gram-negative bacteria (82). Next to this role, NLRC4 also participates in autoimmune inflammatory responses by contributing to TLR activation (144). However, literature data on the influence of PUFA metabolites on the NLRC4 pathway is very limited. It has been shown that 4-HNE does not affect NLRC4 activity and associated IL-1β release but has an inhibitory effect on the NLRP3 inflammasome (121). However, so far, there is no direct literature data on the effect of reactive aldehydes (MDA, 4-HNE) on the regulation of the NLRC4 pathway (**Figure 7**).

Regarding eicosanoids, a study in intestinal epithelial cells using C57BL/6J mice and *Salmonella typhimurium* infection revealed NLRC4-mediated production of eicosanoid lipid mediators, showing a significant increase in the level of proinflammatory PGE_2_ (**Figure 7** **),** which promotes vascular leakage and fluid accumulation in the intestinal lumen (145). The same study also suggested the role of caspase-8, in NLRC4 inflammasome responses *in vivo*, as a resistance against bacterial pathogens which can inhibit caspase-1 (145). Caspase-8 has been previously shown to bind the ASC-PYD domain and is involved in ASC speck formation upon *Salmonella* infection (145). The NLRC4 inflammasome over-activation has been indicated as a critical element of cell death, dictating mouse death due to *Salmonella* infection. It has been proposed that “cytokine or eicosanoid storm” is not essential for the FlaTox (selective activator of NLRC4)-induced animal death (146). Moreover, it has been shown that the generation of eicosanoids (such as PGD_2_ and thromboxane B_2_, TXB_2_) was almost completely eliminated in Nlrc4^−/−^ mice, except PGE_2_ (146). Together with that, their pro-inflammatory signaling roles associated with autocrine or paracrine signaling may still play an important role in the determination/generation of cellular response against such infection, such as in the case of apoptosis as a backup pyroptosis (146). Indeed, PGE_2_, produced due to bacterial pathogen infection, was found to enhance/prolong inflammasome activation, running the generation of pore-induced intracellular traps, and ultimately preventing bacterial escape from the dying cell (30).

Caspase-1-mediated arachidonic acid release may participate in PGE_2_ generation (85). Proteomic studies suggest that the signal that emerged in response to NLRC4-mediated caspase-1 activation may play a role in this situation (85). Therefore, the relationship between PGE_2_ production and NLRC4 activity is still an open topic for research. Although NLRC4 activity has been observed to induce PGE_2_ production, the question remains whether this production has a feedback effect on NLRC4, potentially as in the case with NLRP3. In addition to the specific involvement of NLRC4 in the development of bacterial infection, research is still ongoing on the involvement of NLRC4 in the inflammatory response observed in autoimmune diseases. However, this requires specification of the participation of PUFA metabolites both in the inflammatory activity of NLRC4 and in the cellular response associated with this activity, especially at the proteomic level in the setting of infections/autoinflammatory diseases. This includes, among others: the assessment of mitochondrial dysfunction and the associated apoptotic response (Bcl2, Bax) and the interaction of oxidative stress with inflammation (Nrf2-NF-κB interplay), as well as ER stress on NLRC4 activity pathways. Also, the analysis of changes in activating transcription factor 6α (ATF6), PRKR-like ER kinase (PERK), and inositol-requiring enzyme 1 (IRE1)/X box-binding protein 1 (XBP1) profiles could reveal the contribution of differences in lipid metabolism related to physiological synthesis of fatty acids and their β-oxidation as well as activation of PPARα (IRE1/XBP1) and regulation of glucose and lipid metabolism (PERK/eukaryotic initiation factor 2α, eIF2α) (125).

In addition to bacterial infection, the involvement of NLRC4 in the inflammatory response in autoimmune diseases continues to be explored, including the involvement of mentioned lipid metabolites in modulating both the inflammatory activity of NLRC4 and the cellular response associated with this activity. It is suggested that the relationship between mitochondrial dysfunction and the associated apoptotic response (Bcl2, Bax) and the interaction of oxidative stress with inflammation (Nrf2-NF-κB interaction), including the relationship with ER stress and the formation of protein aggregates, may expand knowledge on about the NLRC4 pathway. In addition to ongoing research on NAIP/NLRC4 mutations contributing to both bacterial infections and sterile inflammation in autoimmune and inflammatory diseases (84), the interplay between NLRC4 and PUFA metabolites may open another window for developing new therapeutic strategies.

### 4.4 The AIM2 inflammasome

AIM2 presents essential activities in inflammatory response against pathogens, but also in autoimmunity in both inflammasome-dependent and -independent roles such as sensing the micronuclear DNA to trigger inflammasome responses in case of genotoxic stress and cell cycle dysregulation, as suggested before (86). So far, its ability to bind dsDNA from the host’s damaged cells is attracting attention regarding a potential alarmin receptor activity (147). Even nowadays, regarding to essential roles of AIM2 in the condition of autoinflammatory conditions and cancer due to the potential recognition of DNA-RNA hybrids by AIM2 and its activation, AIM2 is an important research point for immunotherapeutic approaches (148).

AIM2, as a DNA sensor, has been found to promote macrophage activation and differentiation by recognizing syngeneic lymphocyte-derived apoptotic DNA (apopDNA) in systemic lupus erythematosus (149). Moreover, AIM2 knockdown was able to reverse apopDNA-induced macrophage activation (149). However these data did not demonstrate a direct involvement of lipid metabolism in AIM2 activity, it is indicated that lipid metabolism is involved in the activation of both M1 and M2 macrophages, although the exact mechanism has not been elucidated (150). Activation of peroxisome proliferator-activated receptor (PPARγ) and proliferator-activated receptor coactivator 1β is known to mediate the transcription of M2 signature genes after stimulation with oleic acid and IL4 (150). Furthermore, the major role of macrophages in sterile inflammation or removal and neutralization of targets is mediated by PRRs by displaying oxidation-specific epitopes (151). Therefore, although not directly established, given the role of lipid metabolism, associated with PUFA metabolism, in macrophage activity and polarization, it may be also indirectly linked to AIM2 activity.

**Figure 8:**
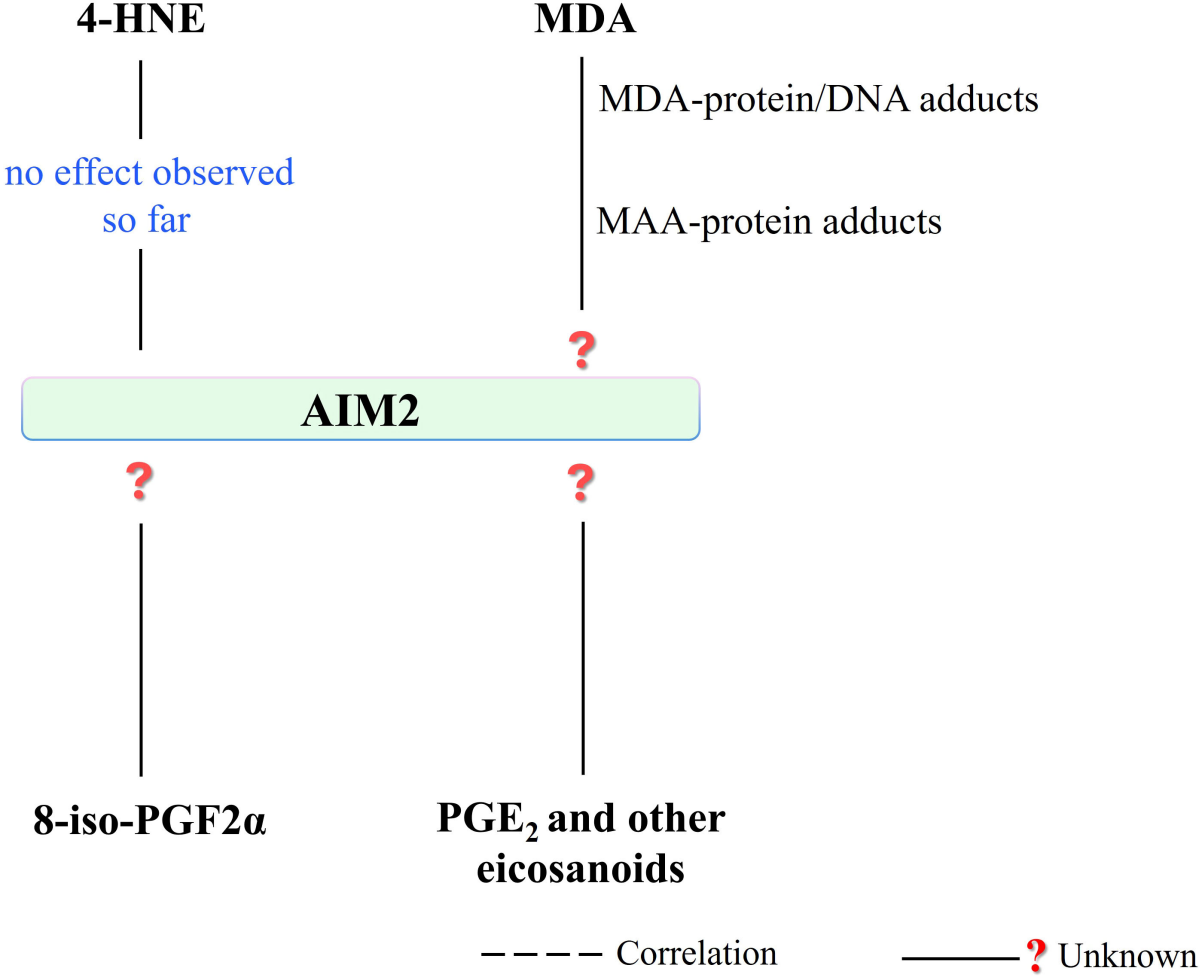
There is no observed effect in the literature regarding the 4-HNE effect on AIM2 activity, so far. In addition, the effect of other PUFA metabolites, which are seen to affect other inflammasomes (NLRP1 and NLRP3), on AIM2 is a subject open to research. (4-HNE, 4-hydroxy-2-nonenal; MDA, malondialdehyde; MAA, malondialdehyde-acetaldehyde modified; AIM2, absent in melanoma 2; 8-iso-PGF2α, 8-iso prostaglandin F 2α; PGE_2_, prostaglandin E2).

Regarding the potential influence of reactive aldehydes on AIM2 activity, the study in mouse models of acute lung injury and sepsis revealed that the use of 4-HNE or increasing endogenous 4-HNE levels by inhibiting glutathione peroxidase 4 activity, independently of Nrf2 and NF-κB signaling, was able to reduce the activation of the NLRP3 inflammasome together with no effect on neither the NLRC4 nor AIM2 (121). This may indicate that the action of 4-HNE is mainly related to the regulation of the NLRP3 inflammasome response. Moreover, another study using MPO^+^ leukocytes obtained from vehicle-treated mice for postoperative intestinal obstruction (POI) showed that late-phase IL-1β release is dependent on the AIM2 inflammasome, with no obvious signs of oxidative stress during POI (152). This study showed a time-dependent increase in SOD activity and the DNA oxidation product - 8-OH-2-deoxyguanosine. Consequently, these data may also support the lack of a significant effect of 4-HNE on AIM2 activation (**Figure 8**).

Interestingly, only one recent study examining the antioxidant/anti-inflammatory effects of demethylene berberine (DMB), a berberine derivative, on AIM2 inflammasome activity in the setting of acute *Pseudomonas aeruginosa* pneumonia found that DBM was able to ameliorate the effects induced by *P. aeruginosa.* In contrast, inflammasome activity AIM2 simultaneously showed a dose-dependent decrease in MDA level (153). Literature data on direct or indirect interference of MDA with the AIM2 pathway are very limited. Therefore, there is still an unsolved problem requiring experimental explanation regarding the assessment of the impact of the formed protein adducts with MDA on the activity of the AIM2 inflammasome (38), as well as the formation of DNA-MDA adducts and related changes in gene expression along with epigenetic issues (154, 155).

It has been indicated that highly immunogenic MDA/acetaldehyde adducts formation with proteins (MAA-protein adducts formed via the breakdown of acetaldehyde and the covalent interaction of two aldehydes and proteins/lipoproteins) appears to promote triglycerides accumulation and progression of the inflammatory response in endothelial cells by modulating cellular metabolism (156). The pro-inflammatory action of MAA adducts (HSA-MAA, human serum albumin modified by MAA and LDL-MAA, low-density lipoprotein modified by MAA), in endothelial (CRL 2167) and macrophage (J774) cell lines was presented by demonstrating an increase in cytokine response (of IL-6, TNF-α, and IL-1β) (157). Although these examples do not provide direct information specifically about inflammasome complexes, their contribution to the formation of inflammatory responses is clear. Thus, evaluating the effect of MAA adducts on both AIM2 and other inflammasomes through changes in both DNA and protein levels may also support understanding the complex molecular signaling behind the inflammasome activity. Especially, regarding the DNA sensing ability of AIM2 and recognition of DNA-RNA hybrids by AIM2 mentioned above, it is important to evaluate the effect of any potential changes in DNA structure due to MDA and/or MAA interaction (158) on AIM2 activity.

Despite the lack of literature data showing a direct relationship between AIM2 activity and 8-isoprostanes, it was indicated that the level of 8-iso-PGF2α in serum (159) and AIM2 (160) positively correlates with the severity of the condition of patients with community-acquired pneumonia (CAP). Moreover, it has been also speculated that 8-iso-PGF2α may play a detrimental role in the pathophysiology of CAP (159). Thus, the potential interference of 8-iso-PGF2α in AIM2 activation/co-activation may be a target for developing therapeutic strategies against CAP pathophysiology. Thus, the potential interference of 8-iso-PGF2α in AIM2 activation/coactivation may be a target for the development of therapeutic strategies against CAP pathophysiology, in which increasing cAMP levels by 8-iso-PGF2α and inhibiting platelet function has been suggested (161). Moreover, the AIM2 inflammasome antagonism of type I interferon signaling – crosstalk between AIM2 inflammasome and cyclic GMP-AMP synthase pathway activating type I IFN expression – has been suggested during not only pathogen infection but also sterile inflammation (86). Therefore, AIM2 activity, interacting with the cyclic GMP-AMP synthase pathway (86), should also be evaluated in connection with 8-iso-PGF2α in this respect.

Besides, studies on peripheral blood mononuclear cells from patients with chronic obstructive pulmonary disease (PBMC) showed that the AIM2 inflammasome-dependent release of IL-1α was not related to the release of eicosanoids, as it did not show an increase in PGE_2_ levels in the case of AIM2 activation (162). Moreover, *Francisella tularensis*-mediated suppression in the AIM2 inflammasome activation has been shown in bone marrow-derived macrophages obtained from mice by demonstrating a decrease in AIM2-dependent IL-1β level (163). Together with that, the overproduction of proinflammatory PGE_2_ was observed in a lethal murine *Francisella novicida* infection model (164). Along with the ongoing research associated with the determination of the AIM2-dependent eicosanoid generation induced by *Francisella* infection (165), there is a research need for the estimation of the potential role of the prostaglandins, such as PGE_2_ mentioned here, and other eicosanoids in the regulation of both cell- and infection-specific AIM2 activity.

It was found that due to the suppressive effect of AIM2 on colorectal cancer, the AIM2 inflammasome plays a role in preventing the development of this cancer (AIM2 effect in DNA-dependent activation of Akt regulating epithelial cell proliferation via protein kinase) (166). An omics study involving single-cell analysis showed the overall survival of colorectal cancer patients with low AIM2 was significantly lower than that in the group with high AIM2 expression (167). These studies also mentioned a dysregulated eicosanoid profile, which may modify the process of colon inflammation and carcinogenesis (168). PGE_2_, whose signaling involves chronic inflammation in the tumor microenvironment, has been found to associate with tumorigenesis of colon cancer, promoting tumor growth (169). Therefore, taking into account epigenetic factors reflecting the individual metabolic differences of patients with colon cancer, further studies on the direct/indirect involvement of eicosanoids (including PGE_2_) in the regulation of AIM2 activity would be helpful in the development of targeted therapies against colon cancer. Also, regarding PGE_2_, a recent study indicates the relationship between AIM2 upregulation and activation of the TNFα-NF-κB signaling by showing upregulated AIM2-mediated IL-1β secretion and activation of STAT1/NF-κB-related pathway in oral squamous cell carcinoma cells (170). Thus, the possible association of PGE_2_ with AIM2 activity may also be possible due to the effect of PGE_2_ on NF-κB signaling (139, 140).

It is clear that AIM2, like the other inflammasomes mentioned above, is a therapeutic target in a wide range of diseases, such as inflammatory diseases including cardiovascular disease (171), cancer, and infectious diseases (172). In addition to what we know so far, highlighting the DNA sensor role of AIM2, further analysis of the impact of PUFA metabolites on AIM2 activity may provide different perspectives for therapeutic approaches targeting AIM2, especially in cancer accompanied by complex metabolic alterations (173).

## 5 Metabolic signaling pathways affected by PUFA metabolites and their impact on inflammasome activities

Due to the involvement of cellular metabolism in energy homeostasis, cell growth, and proliferation, adaptation to environmental changes, and disease states, it is among the key biochemical processes analyzed in targeted pharmacotherapeutic approaches (174). However, PUFA metabolites significantly influence among others: metabolic signaling pathways regulating inflammation, including inflammasomes, and the most important signaling pathways in this regard are discussed below.

### 5.1 NF-κB and PPAR pathway

The primary signaling pathway affected by PUFA metabolites is NF-κB signaling, which can be viewed as a key regulator of the NLRP3 pathway, and the interaction of 4-HNE with this molecular signaling is believed to be of greatest importance. However, 4-HNE is known to be a critical regulator of NLRP3 activity, both through direct molecular interaction (and subsequent inhibition of NEK7) and interference with NF-κB and Nrf2 crosstalk. However, a candidate for forming an adduct with 4-HNE is the FIIND domain of NLRP1, due to its structure with redox-sensitive amino acids.

The key element in the influence of PUFA metabolites on inflammasome activity seems to be changes in signaling related to Nrf2, NF-κB, and their interactions. This includes, among others: direct 4-HNE-mediated structural changes and associated changes in inhibition of the NF-κB pathway and inactivation of the antiapoptotic Bcl-2 (92). Moreover, PGE_2_ (via the EP2/EP4-cAMP-PKA-CREB axis) (139) and 15d-PGJ2, by enhancing the transcriptional activity of PPAR-γ through inhibiting the NF-κB and JAK-STAT pathways (116), can alter the activity all inflammasomes (both NLRP1, NLRP3, NLRC4 and AIM2). In contrast, the feedback mechanism between PGE_2_ and NLRP3 levels, through modulation of NF-κB signaling depending on the intensity of 4-HNE production, may play an important regulatory effect in modulating chronic inflammation caused by oxidative stress. In fact, increased 4-HNE generation can also trigger a cellular response to oxidative stress with a synergistic response to ferroptosis and pyroptosis (112) in association with NLRP1 (potentially).

Furthermore, increased generation of 8-iso-PGF2α (together with an increase in 4-HNE generation accompanied by Nrf2 dysregulation in case of aging-related oxidative stress) has been also found to be associated with promoting NF-κB signaling (175). 8-iso-PGF2α can also regulate NLRP3 activity by increasing cAMP (161). Here, the increase of cAMP may act as a regulatory point on the 8-iso-PGF2α-mediated NLRP3 activity, associated with NF-κB signaling (cAMP-PKA-NF-κB axis) (176). Moreover, the cell type- as well as microcellular environmental-dependent actions of cAMP (176), are important for targeted anti-oxidant and anti-inflammatory approaches modulating inflammasome activities (especially in the case of NLRP3).

Therefore, PUFA metabolites, which are agonists of the PPARs receptor have an important therapeutic significance, especially in the treatment of diseases such as inflammation and cancer (177). It has been suggested that NF-κB binding to DNA may be inhibited by PPAR (thiazolidinedione) agonists (177). It was also highlighted that 15d-PGJ2 could inhibit the expression of metalloproteinase-9 via NF-κB and AP-1 and modulate breast cancer invasion through the PPARγ/HO-1 signaling pathway (178). Therefore, regarding the inhibition of NLRP1 and NLRP3 via 15d-PGJ2-mediated PPARγ activation (103), the modulation of PPARγ-NF-κB signaling should also be considered as an indirect regulation of NLRP1 and NLRP3 activity by 15d-PGJ2. However, it is not known whether the severity of oxidative stress (changes in 15d-PGJ2 levels) plays a role in modifying this effect.

### 5.2 SREBP pathway

Another molecular pathway modulated by lipid metabolism products is sterol regulatory element binding protein (SREBP) signaling, which involves the biosynthesis of triglycerides, fatty acids, and also cholesterol (179). However, it has been shown that SREBP1a not only activates lipogenesis in macrophages but also induces *Nlrp1a* gene encoding (150, 180). Furthermore, SREBP1a deficiency (in mice) has been found to be associated with a defective innate immune response and LPS-mediated inhibition of lipid biosynthesis (180). Moreover, recent findings suggest that SREBP cleavage activating protein (Scap)-SREBP1 protease (S1P)/S2P can promote the phosphorylation and subsequent activation of NF-κB through the release of IκBα for IκB kinase (Ikk) (181). In contrast, TNFα-mediated NF-κB activation promotes activation of SREBP2 and SREBP2-dependent gene expression (182). Furthermore, placental exposure to 4-HNE was found to promote the expression of the genes related to lipogenesis and lipid uptake, while 4-HHE (4-hydroxy-2-hexenal) decreased the expression of the genes related to lipogenesis and lipid uptake (SREBP1 and SREBP2) (183). However a direct link between SREBP and 4-HNE is not yet known, it is known that the activity of both NLRP3 and NLRP1 is mediated - correlatively - by the SREBP pathway, as well as by 4-HNE. This further highlights the key role of lipids in regulating inflammasome activity.

### 5.3 AMPK pathway

Another key signaling that regulates energy homeostasis during metabolic stress, in which reprogramming AMPK metabolism has great potential, as a target for the treatment of inflammatory diseases, is AMP-activated protein kinase (AMPK) signaling (184). It inhibits NF-κB signaling and reduces the accompanying inflammatory damage cells, acting as a negative regulator of inflammation (185). An enhanced antioxidant response due to AMPK activation has also been demonstrated (186). Furthermore, activation of AMPK and overactivation of the NLRP3 inflammasome (186) were demonstrated in blood cells from fibromyalgia patients, which promoted increased levels of IL-1β, IL-6, TNF-α, iNOS, and COX2 in AMPK knockout mice (187). Despite the lack of data on eicosanoids, it should be noted that the eicosanoid profile in inflammatory conditions may change (related to the inflammatory response) as a result of changes in COX2 activity. Moreover, 4-HNE can inhibit liver kinase B1 (LKB1), which phosphorylates the α subunit of AMPK and consequently AMPK signaling (188). Therefore, 4-HNE-dependent AMPK signaling can also be considered as another element in the modulation of NLRP3 activity. However, the eicosanoid profile, which may be modified by the above-mentioned changes in the level and/or activity of COX2 and the intracellular redox state, may also act as an additional modulatory axis of NLRP3 activity.

Furthermore, AMPK/mTOR-mediated NLRP1 activation and autophagy dysfunction are associated with β-amyloid (Aβ) peptides in APP/PS1 9M mice (189) while NLRP1 down-regulation causes a significant decrease in phosphorylated AMPK levels (189), and metformin - via AMPK signaling - causes attenuation of NLRC4 activation in acute lung injury in mice (190). The possibility of AIM2 activation via end-binding to protein 1, regulated by AMPK, has also been suggested (191). In contrast, a study in mice shows that the increased free PUFA levels and dysregulated eicosanoid profile resulting from a high-fat diet are reversed by AMPK activation (192). Therefore, PUFA metabolites, whose production is altered by intracellular redox signaling, may also participate in the regulation of inflammasome activity in association with AMPK signaling, which is activated by ω-3 (DHA) and ω-6 (LA) PUFAs (193, 194).

### 5.4 mTOR pathway

Mammalian target of rapamycin (mTOR) - a serine-threonine protein kinase - signaling involves critical molecular signaling related to cell stress, growth, proliferation, and metabolic reprogramming (195). In addition, it has been revealed that ω3-PUFA, DHA, can enhance LKB1 signaling which its expression increases leading to AMPK phosphorylation and mTOR inhibition in HeLaS3 cells (196). These data suggest anti-carcinogenic properties of ω3-PUFA associated with the inhibition of mTOR activity as well as tumorigenic cellular metabolism mediated by tumor suppressor activity of LKB1 (196). On the other hand, regarding the connection between oxidative stress and Alzheimer’s disease progression, mTORC1 activation is found to have a strong association with neurodegeneration, ROS-mediated molecular damage, and Alzheimer’s disease neuropathology (197). Moreover, increased 4-HNE generation as well as 4-HNE-protein adducts levels are known in brain regions associated with histopathological alterations in Alzheimer’s disease (197). It has been revealed that 4-HNE can induce mTOR pathway activation (198).

In addition, 15-F_2t_-isoprostane-mediated inhibition of BAC1.2F5 macrophage spreading and adhesion through phosphorylation of Akt which is a component of mTORC2 signaling has been shown (199). Eicosanoid-mediated activation of mTOR signaling is also critical for the regulation of inflammation response and associated cell survival dynamics. PGE_2_ has been shown to induce Y box-binding protein 1 expression via activation of EP1-proto-oncogene tyrosine-protein kinase Src-epidermal growth factor receptor (EGFR)-p44/42 MAPK-mTOR pathway which increases the invasive ability of hepatocellular carcinoma cells (200). Furthermore, it has been revealed that 15(S)-HETE can stimulate angiogenesis through the activation of PI3K-Akt-mTOR-the mTOR substrate S6 kinase 1 (S6K1) signaling (201). Also, a significant reduce in endometrial cancer progression has been demonstrated through the dual inhibition of COX-2 and mTORC1 signaling (202).

Therefore, the regulation of mTOR signaling due to PUFA metabolites appears as one of the key mechanisms due to mTOR-mediated inflammation affecting the tumor microenvironment. In addition to the direct effects of PUFA metabolites (as seen in the case of 4-HNE due to adduct formation), they indirectly change inflammasome activities through mTOR signaling, which may have a significant effect on the modulation of cancer development or progression. Over and above, inflammatory response mediated by mTOR signaling has been highlighted in tumor immune microenvironment by promoting immune cell recruitment (203). And, deregulated mTOR signaling in cancer has been revealed to affect tumor immune microenvironment (203). Thus, potentially, modulation of the activity of NLRP1 and NLRP3, due to the effect of PUFA metabolites (majorly, 4-HNE, 8-isoprostanes, and PGE_2_) on mTOR signaling, appears as a prominent pharmacotherapeutic target, especially in the case of cancer therapies. Besides, mTORC1- and hexokinase 1-dependent glycolysis mediated by TLR ligation has been shown as an essential component of NLRP3 inflammasome (204). Inhibition of NLRP1 activity mediated AMPK/mTOR related-autophagy dysfunction has been suggested (189). Furthermore, a recent study on acetaminophen-induced liver injury in aged mice showed that AIM2 was overexpressed when the expression levels of p62, phosphorylated beclin 1, and phosphorylated mTOR were significantly reduced, suggesting an enhancement of the AIM2-mediated autophagy pathway (205).

### 5.5 GPR120 pathway

The last signaling pathway in this regard is G protein-coupled receptor 120 (GPR120) signaling. It has been shown to regulate inflammation and apoptosis (206). Free fatty acid receptor 4 (FFAR4, also known as GPR120) has been identified as the primary receptor of ω-3 PUFA (206). FFAR4 agonism has been shown to repress NF-κB and associated TNF-α, IL-1, IFN-γ, IL-6, and IL-12 generation via upregulation of PPARγ signaling (206). It has been known that EPA and DHA can alter inflammatory gene expression via binding to GPR120 and PPARγ, and they can give rise to anti-inflammatory and inflammation-resolving mediators called resolvins, and protectins (207). Even, FFAR4 inhibitory effect on NLRP3 activity, due to PPARγ signaling, as well as associated reduction in metabolic inflammation have been indicated (206). On the other hand, GPR120-mediated PI3K/Akt–NF-κB signaling has been indicated as an important angiogenic switch promoting angiogenesis and tumor growth in human colorectal carcinoma (208). Moreover, DHA-mediated GPR120 signaling has been not only shown in the suppression of NF-κB and inhibition of NLRP3 activity but also found in NAIP5/NLRC4 and AIM2 inflammasome activities (209).

Additionally, a recent study in Sertoli cells showed that 12-hydroxyeicosapentaenoic acid (12-HEPE) can induce expression of bone morphogenic protein 4 (BMP4) via GPR120-ERK1/2 activation and protect spermatogonia (210). Another study in a nonalcoholic steatohepatitis mouse model showed that 4-HNE can promote calpain activation via GPR120 signaling, and this results in lysosomal membrane permeabilization and cell death (211). Thus, PUFA metabolism-dependent GPR120 signaling, altering NF-κB signaling and inflammasome activities (mostly NLRP3), appears as a critical regulatory signaling pathway for inflammatory response.

## 6 Conclusion

**Figure 9:**
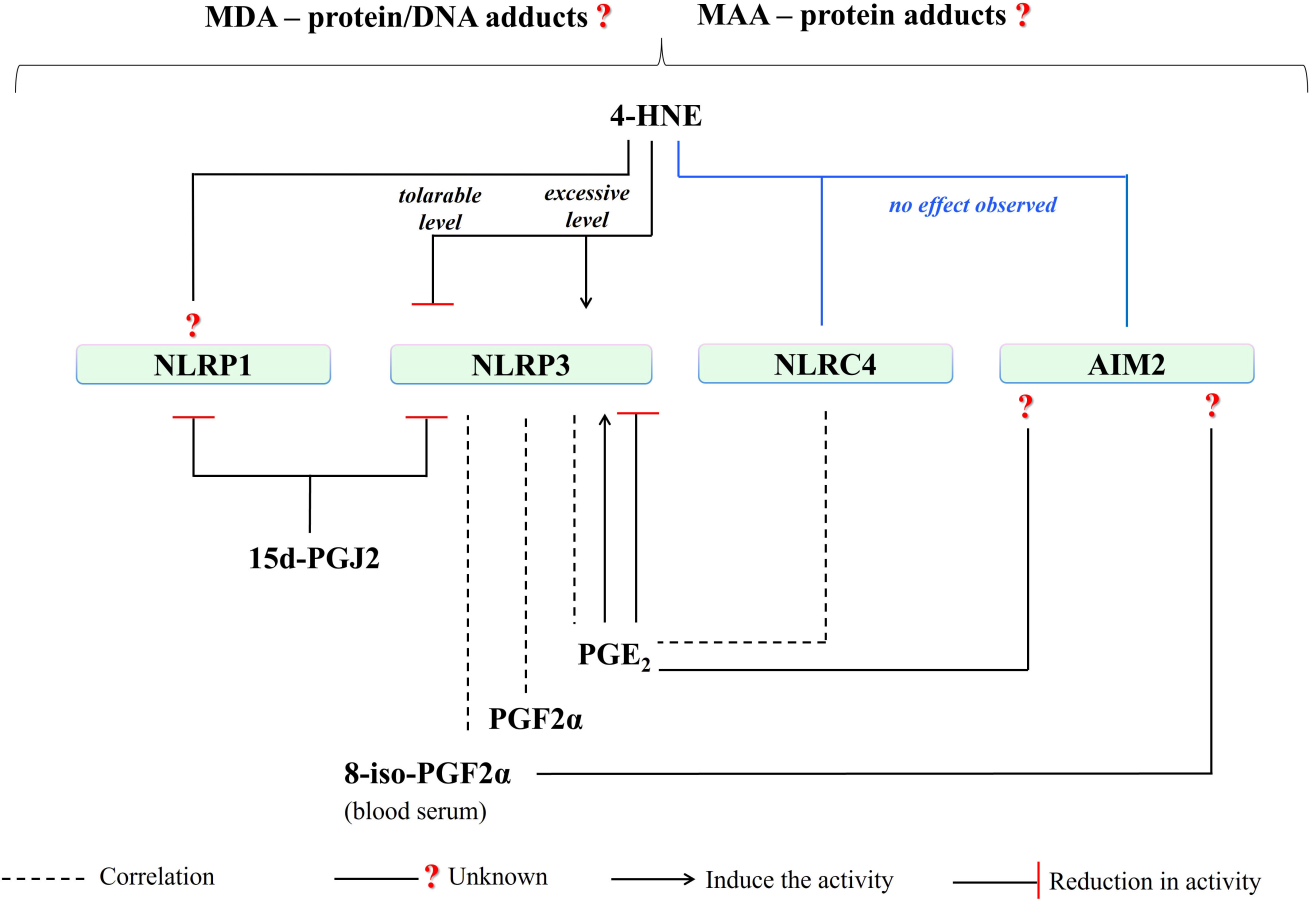
A summary of the influence of PUFA metabolites on activities of well-known inflammasomes. (4-HNE, 4-hydroxy-2-nonenal; MDA, malondialdehyde; NLRP1, NLR family pyrin domain-containing protein 1; NLRP3, NLR family pyrin domain-containing protein 3; NLRC4, NLR family CARD domain-containing protein 4; AIM2, absent in melanoma 2; 15d-PGJ2, 15-deoxy-∆-12,14-prostaglandin J2; PGE_2_, prostaglandin E2; PGF_2a_, prostaglandin F2α; 8-iso-PGF2α; 8-iso Prostaglandin F 2α).

Due to the high susceptibility of lipid species to oxidative modifications, lipid metabolism turns out to be an important variable in chronic inflammation associated with oxidative stress. PUFA metabolites play a key role in the development and progression of inflammation, by interfering with the activity of inflammasomes (**Figure 9**). Among the reactive aldehydes, 4-HNE seems to be a critical regulator, especially of NLRP3 activity, which also has a great potential to modulate NLRP1 activity through direct interaction with its FIIND domain. However, questions regarding MDA-mediated changes in inflammasome activity remain open. Moreover, the potential impact of 8-iso-PGF2α on AIM2 is a matter of interest associated with the pathophysiology of CAP. Regarding eicosanoids, prostaglandin 15d-PGJ2 was found to inhibit the activity of both NLRP1 and NLRP3. However, PGE_2_ appears to be positively correlated with both NLRP3 and NLRC4, and the possibility of a positive or negative effect of PGE_2_ on NLRP3 activity seems to be of great metabolic interest. However, these effects may vary depending on the diversity of triggering factors and cell specificity, as well as the severity of oxidative stress. Moreover, the impact of PUFA metabolism on the modulation of critical metabolic signaling pathways mainly involves NF-κB, PPAR, SREBP, AMPK, mTOR, and GPR120 signaling, and appears as an indirect/auxiliary regulation, potentially, in the alteration of inflammasome activities. This situation is especially obvious in the regulation of NLRP3 activity regarding to mentioned signaling pathways.

Since the above analysis concerned the influence of PUFA metabolites on the activity of inflammasomes, an attempt was made to limit the considerations to showing only well-characterized inflammasomes. This narrowed the problem while indicating expectations for future studies, which should demonstrate an inflammasome response associated with organ/cell specificity and/or specificity for a particular pathological condition. This would also allow us to understand the multidirectional aspects of the biological actions of PUFA metabolites related to their chemical structure as well as the location of the inflammasomes as cell/organ-specific. Moreover, it can be hoped that further research will contribute to expanding knowledge about the interactions of PUFA metabolites with inflammasomes, for instance, in the context of constantly developing antioxidant therapies. These therapies are still not satisfactory concerning the oxidative stress-inflammatory axis, and yet often, but only fragmentarily, perceived in various disease states.

## Glossary

NO: nitric oxide
12-HEPE: 12-hydroxyeicosapentaenoic acid
15d-PGJ2: 15-deoxy-∆-^1214^-prostaglandin J2
4-HNE: 4-hydroxy-2-nonenal
AA: arachidonic acid
Aβ: β-amyloid
AIM2: absent in melanoma 2
Akt: serine/threonine-specific protein kinase
ALA: linolenic acid
ALDH2: aldehyde dehydrogenase 2
AP-1: activator protein-1
AMPK: AMP-activated protein kinase
apopDNA: apoptotic DNA
APP/PS1: amyloid precursor protein/presenilin
ASC: apoptosis-associated speck-like protein containing CARD
ATF6: activating transcription factor 6α
Bax: B-cell lymphoma protein 2 (Bcl-2)-associated X
Bcl-2: anti-apoptotic B-cell lymphoma-2
BMP4: bone morphogenic protein 4
cAMP: cyclic adenosine monophosphate
CAP: community-acquired pneumonia
CARD: caspase recruitment domain
CARD8: a caspase-1-activating protein having just FIIND domain and CARD domain
CLRs: C-type lectin receptors
COXs: cyclooxygenases
CREB: cyclic AMP-Response Element Binding Protein
Cu²⁺: copper ion
CYP450s: cytochromes p450
cyPGs: cyclopentenone prostaglandins
DAMPs: damage-associated molecular patterns
DHA: docosahexaenoic acid
DMB: demethylene berberine
DPP8/DPP9: dipeptidyl peptidases 8 and 9
dsDNA: double-stranded DNA
eIF2α: eukaryotic initiation factor 2α
EGFR: epidermal growth factor receptor
EP4: PGE_2_ receptor subtype 4
EPA: eicosapentaenoic acid
ER: endoplasmic reticulum
ERK: extracellular regulated kinase
Fe²⁺: ferrous iron
FFAR4: free fatty acid receptor 4
FIIND: function-to-find domain ZU5-UPA domain
GBPs: guanylate-binding proteins
GPX4: glutathione peroxidase 4
GPR120: G protein-coupled receptor 120
GSDMD: gasdermin D
GSH: glutathione
H_2_O_2_: hydrogen peroxide
HETEs: hydroxyeicosatetraenoic acids
HIN: hematopoietic
interferon-inducible: and nuclear localization
HODEs: hydroxyoctadeca-dienoic acids
HSA-MAA: human serum albumin modified by malondialdehyde-acetaldehyde
IKK: IκB kinase
IKKβ: IκB kinase β
IL‐18: interleukin 18
IL‐1β: interleukin 1β
IL-8: interleukin 8
iNOS: inducible nitric oxide synthase
IRE1: inositol-requiring enzyme 1
IκBα: inhibitory κB-α
JAK: janus kinase
JNK: c-Jun N-terminal kinase
LA: linoleic acid
LDL-MAA: low-density lipoprotein modified by malondialdehyde-acetaldehyde
LKB1: liver kinase B1
LOXs: lipoxygenases
LPS: lipopolysaccharide
LRRs: leucine-rich repeats
LTs: leukotrienes
LXs: lipoxins
MAA: malondialdehyde-acetaldehyde modified
MAPK: mitogen-activated protein kinase
MDA: malondialdehyde
mTOR: mammalian target of rapamycin
MyD88: myeloid differentiation primary response 88
NACHT: a central nucleotide-binding and oligomerization domain (NOD)
NAIP: NLR family apoptosis inhibitory protein
NEK7: a member of the NIMA (never-in-mitosis A) related kinases family
NETs: neutrophil extracellular traps
NF-κB: nuclear factor - kappa B
NLR: nucleotide-binding domain leucine-rich repeat containing
NLRC4: NLR family CARD domain-containing protein 4
NLRP1: NLR family pyrin domain-containing protein 1
NLRP3: NLR family pyrin domain-containing protein 3
NLRs: nucleotide-binding oligomerization domain-like receptors
Nrf2: nuclear factor 2 associated with erythroid 2
O_2_^•−^: superoxide anion
O_3_: ozone
ONOO⁻: peroxynitrite
p38: a member of mitogen-activated protein kinases (MAPKs)
p65: RelA a heterodimer in the NF-κB signaling pathway
PAL: palmatine
PAMPs: pathogen-associated molecular patterns
PBMC: patients with chronic obstructive pulmonary disease
PERK: PRKR-like ER kinase
PGE_2_: prostaglandin E2
PGF_2α_: prostaglandin F2α
PGs: prostaglandins
PI3K: phosphatidylinositol 3-kinase
PKA: protein kinase A
PKD: protein kinase D
PLA: phospholipase A
POI: postoperative intestinal obstruction
PPARα: peroxisome proliferator-activated receptor alpha
PPAR-γ: peroxisome proliferator receptor-γ
Pro-caspase-1: immature caspase 1
Pro-IL-18: immature interleukin 18
Pro-IL-1β: immature interleukin 1β
PRRs: pattern recognition receptors
PTMs: post-translational modifications
PUFA: polyunsaturated fatty acid
PYD: pyrin domain
PYHIN: the human pyrin and hematopoietic interferon-inducible nuclear (HIN) domain
RLRs: RIG-I-like receptors
RNS: reactive nitrogen species
ROS: reactive oxygen species
S6K1: the mTOR substrate S6 kinase 1
SOD: superoxide dismutase
SREBPs: sterol regulatory element-binding proteins
STAT: signal transducers and activators of transcription
TLRs: toll-like receptors
TNF-α: tumor necrosis factor alpha
TRPV4: transient receptor potential cation channel subfamily V member 4
TRX1: thioredoxin-1
TXB2: Thromboxane B_2_
TXs: thromboxanes
UBR2: E3 ubiquitin transferase
XBP1: X box-binding protein 1
ω-3: omega-3
ω-6: omega-6
